# Physiologically Based Pharmacokinetic Model of Magnesium Implant Absorption and Distribution in Tissue and Organs

**DOI:** 10.1021/acsomega.5c06910

**Published:** 2026-01-22

**Authors:** John P. Ward, Safia K. Ahmed, Yang Liu

**Affiliations:** † Department of Mathematical Sciences, 5156 Loughborough University, Loughborough LE11, U.K.; ‡ Centre of Biological Engineering, Wolfson School of Mechanical, Electrical and Manufacturing Engineering, Loughborough University, Loughborough LE11 3TU, U.K.

## Abstract

The long-term accumulation of magnesium (Mg­(II)) ions in human patients resulting from the biodegradation of clinical Mg (alloy) implants is investigated using a physiologically based pharmakinetic (PBPK) mathematical model. In severe cases, an excess of Mg in blood (hypermagnesemia) causes a range of health concerns and potentially death. Studies investigating clinical Mg devices generally indicate that there is little risk in healthy patients; however, there is concern that excessive Mg accumulation may occur in patients who are elderly, have osteoporosis, and/or have renal disease. The PBPK model describes the time evolution of Mg concentrations in blood, tissue, and bone compartments in response to Mg sourced from diet and implant(s) devices, over the implant’s lifetime. It predicts that Mg absorption in the tissue and bone compartments is the key factor in modulating long-term serum levels due to their large volume and Mg load. Furthermore, the time scale of observable accumulation can take several months to years, suggesting that for vulnerable patients, the Mg levels should be monitored throughout the lifespan of an Mg implant. Most of the model parameters can be estimated from simple patient measurements; thus, the model is the first step toward a practical patient-specific framework for Mg and for other biodegradable implant devices to inform medical treatments in response to the potential long-term accumulation of biodegraded products.

## Graphical abstract

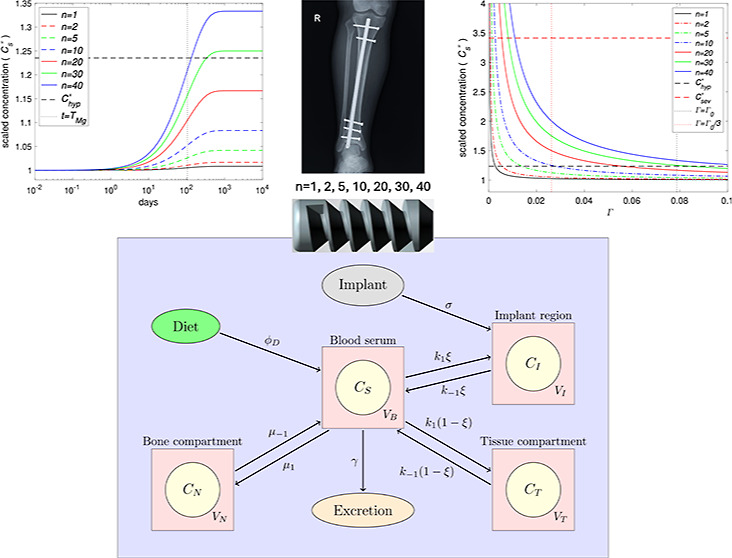

## 1 Introduction

Implant devices made from magnesium (Mg) and its alloys have a number of clinical applications, particularly in bone repair. The devices degrade in aqueous environments over several months allowing the bone to grow as the implant degrades.^1–4^ The corrosion processes of the implant release Mg­(II) ions into the human body that are distributed and absorbed throughout via the bloodstream. While studies suggest that the release of Mg­(II) ions from implants is unlikely to cause medical problems to healthy humans,^5–8^ for individuals with compromised kidney function or certain medical conditions, for example, osteoporosis and renal disease, the long-term release of Mg­(II) ions from implants increases the risk of elevated Mg levels in the body perhaps resulting with hypermagnesemia (i.e., serum Mg concentration >1.05 mmol/L); indeed Walker et al.^9^ proposed that Mg levels in serum should be monitored over the course of an implant’s life to mitigate against such health concerns.

Under normal circumstances, a stable level of magnesium in the body is maintained by three major organs, intestine, kidneys, and bone, whereby in healthy people, serum Mg concentration lies within 0.65–1.05 mmol/L.^10^ Typically, around 25–55% of Mg from food^11,12^ is absorbed into the bloodstream. Mg in the bloodstream is filtered by kidneys, and the healthy kidneys are able to excrete more or less Mg to help preserve homeostatic levels in the blood.^11^ For storage, the Mg­(II) ion is continuously exchanged between the blood and bone, muscle, and other soft tissues.^13^ The effective regulatory processes by the kidney, bone, and gut means that for healthy individuals, extra Mg resulting from a degrading Mg implant is unlikely to pose any risks.^14,15^ This is consistent with findings from the short-term (<2 weeks) *in vivo* studies by Wang et al.^16^ and Sato et al.,^17^ which showed little evidence of Mg increase in serum, faeces, and in a number of organs. Furthermore, there appears to be little difference between healthy rats and those with compromised kidney function.^16,18^ A noticeable increase in Mg content in urine was only observed using implants proportionately much larger than those used clinically.^17^ A longer-term study by Zhang^19^ demonstrated that healing of the bone occurred while the Mg rod degrades, without any noticeable increase in blood levels of Mg. Though these studies seem to suggest that Mg implants are unlikely to cause any significant health issues, there appears to be no long-term study representing the physiology of health-compromised individuals.

More understanding of corrosion behaviors of magnesium implants and change of their strength have been gained, particularly with the application of multiscale modeling to predict long-term effects of corrosion.^20–22^ There are relatively few studies using mathematical models to describe the dynamics of Mg­(II) ions in the body. Simple pharmacokinetic pharmacodynamic (PKPD) models have been proposed to describe the effects of magnesium containing drugs on the cardiovascular response^23^ and on the relationship between plasma concentrations and blood pressure in pre-eclamptic women.^24^ Avioli et al.,^25^ Sojka et al.,^26^ and Sabatier et al.^12^ investigated the kinetics of Mg isotope biomarkers using compartmental, physiologically based pharmacokinetic (PBPK) models, over the period of 1–2 weeks.

The modeling in the current paper also uses a PBPK approach and is aimed at predicting the long-term accumulation of Mg in humans, resulting from the corrosion of single, multiple, and/or large (e.g., for osteosarcoma treatment^27^) implants, with and without dietary control.

- The modeling approach has wider application for clinically relevant biodegradable implant materials
- the first step for a predictive tool for the long-term accumulation of biodegradable products in the body to providing a means of informed, patient specific, medical decision making.

## 2 Experimental Section

### 2.1 Materials and Methods

#### 2.1.1 Mathematical Modeling

Magnesium is present throughout the body, and the proposed PBPK model will track in time the Mg concentration in various bodily compartments, namely, the bone (the quantities for which are denoted with a subscript *N*), blood (including serum *s* and red blood cells), and tissue (*T*, bundling together muscle and soft-tissues, as they have similar Mg concentrations, see Supporting Information B). In addition, a compartment representing the localized tissue in the immediate vicinity of the implant (*I*) is also considered that will initially absorb Mg­(II) ions released from the implant. In Supporting Information A, a model is discussed where in each compartment the Mg is assumed to be “exposed” (i.e., free to be exchanged between compartments) and “unexposed” (i.e., cannot be exchanged between compartments).^13,14,28^ The formulation of this model uses mass action principles applied to the pathway as shown in Figure S1 (as is standard for PBPK models) resulting in a system of linear ordinary differential equations (ODEs). The model takes into account Mg sourced from diet and implant (assuming to occur at a constant rate, for simplicity), blood transport, exchange between the blood and tissue/bone compartments, and excretion. The various processes operate on a broad range of time scales, for example, equilibration of Mg­(II) ion concentrations between red-blood cells and serum (minutes),^13,14^ excretion (hours), and life-span of the implant (months/years). In Section A.1 of Supporting Information A, this model is simplified, without loss of accuracy, in order to focus on events occurring over the time scales of interest, namely, *O*(hours)-*O*(years), to obtain the following system of ODEs for exposed Mg­(II) ion concentrations (*C*_j_ for j = s, N, T, I) in the four compartments

1 $$\left(V_{s}\left(1+\xi_{1}\right)+V_{r}\xi_{2}\right)\frac{dC_{s}}{dt}=\phi_{D}-\left(\gamma +\mu_{1}+k_{1}\right)C_{s}+\mu_{-1}C_{N}+k_{-1}\left(\left(1-\xi \right)C_{T}+\xi C_{I}\right)$$

2 $$\phi V_{N}\frac{dC_{N}}{dt}=\mu_{1}C_{s}-\mu_{-1}C_{N}$$

3 $$V_{T}\left(1+\xi_{3}\right)\frac{dC_{T}}{dt}=\left(1-\xi \right)\left(k_{1}C_{s}-k_{-1}C_{T}\right)$$

4 $$V_{I}\left(1+\xi_{3}\right)\frac{dC_{I}}{dt}=\sigma +\xi \left(k_{1}C_{s}-k_{-1}C_{I}\right)$$

where ξ = *V*_I_/*V*_T_tot__; the pathways represented by this model are shown in Figure 1, and explanations of the model parameters are listed in Table 1, together with data-derived value estimates. In the simulations to follow, the Mg implant(s) is introduced at *t* = 0, whereby the compartmental Mg­(II) ion concentrations are set at homeostatic levels, denoted as

5 $$C_{s}={\mathrm{C̅}}_{s}^{e},,C_{N}={\mathrm{C̅}}_{N}^{e},,C_{T}={\mathrm{C̅}}_{T}^{e},,C_{I}={\mathrm{C̅}}_{T}^{e}$$

where ${\mathrm{C̅}}_{s}^{e}=\phi_{D}/\gamma ,,{\mathrm{C̅}}_{N}^{e}=\mu_{1}{\mathrm{C̅}}_{s}^{e}/\mu_{-1}$ and ${\mathrm{C̅}}_{T}^{e}=k_{1}{\mathrm{C̅}}_{s}^{e}/k_{-1}$ , being the steady-state of the model without the implant (i.e., when σ = 0). The plots in the Section 2.2 will present the concentrations in the normalized form, that is, $C_{j}^{*}=C_{j}/{\mathrm{C̅}}_{j}^{e}$ , for j = s, N, T, and $C_{I}^{*}=C_{I}/{\mathrm{C̅}}_{T}^{e}$ , so that at *t* = 0, the (homeostatic) concentrations are given by (*C*_s_^*^, *C*_N_^*^, *C*_T_^*^, *C*_I_^*^) = (1, 1, 1, 1); this means that the predicted relative changes of the compartmental Mg concentrations can be immediately seen in the graphs.

**Figure 1:**
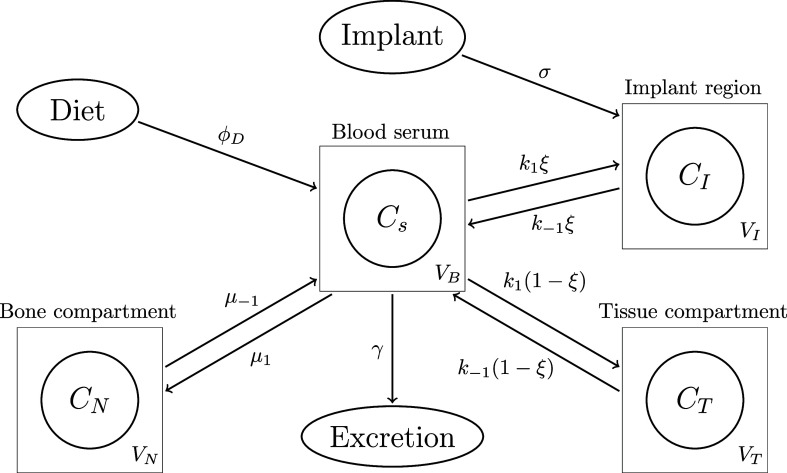
Pathway diagram showing magnesium exchange between the four compartments as described by eqs 1–4. The C_*_ and V_*_ represent the Mg­(II) ion concentration and volumes of the respective compartments, with *V*_B_ = *V*_s_ + *V*_r_, and the rate constants are described in Table 1. The simplification from the more complete pathway system and corresponding model is discussed in Supporting Information Section A.1.

**Table 1:** Values for the Model Parameters for Healthy Individuals Discussed in Supporting Information B ^a^

parameter	description	value	units	source
*V*_s_	volume of the serum compartment	3	L	^13,14^
*V*_r_	volume of RBCs	2	L	^13,14^
*V*_N_	volume of the bone	12.3	L	^13,14^
*V*_T_tot__	volume of tissues	52.7	L	^13,14^
*V*_I_	volume of the implant zone (per implant)	0.00527	L	-
${\mathrm{C̅}}_{s}^{e}$	homeostatic exposed concentration in serum	0.553	mmol/L	R
${\mathrm{C̅}}_{r}^{e}$	homeostatic exposed concentration in RBCs	0.25	mmol/L	R
${\mathrm{C̅}}_{N}^{e}$	homeostatic exposed concentration in the bone	43.1	mmol/L	R
${\mathrm{C̅}}_{T}^{e}$	homeostatic exposed concentration in tissues	2.20	mmol/L	R
*C*_hom_	homeostatic serum concentration $\left({\mathrm{C̅}}_{s}^{e}+{\mathrm{C̅}}_{s}^{u}\right)$	0.85	mmol/L	R
*C*_hyp_	hypermagnesemia threshold conc. (*C*_s_^e^ + *C*_s_^u^)	1.05	mmol/L	^11^
*C*_sev_	severe serum concentration case (*C*_s_^e^ + *C*_s_^u^)	2.9	mmol/L	^11^
σ	Mg(II) implant release rate (e.g., a 3.2 × 32 mm screw)	0.05	mmol/day	V
ϕ_D_	Mg(II) dietary intake rate after intestinal absorption	6	mmol/day	I
γ	excretion rate constant of Mg(II) in the urine	10.9	L/day	H
μ_1_	ERC between blood and bone	6.05	L/day	I
μ_–1_	ERC between bone and blood	0.0775	L/day	H
*k*_1_	ERC between blood and tissue	138	L/day	I
*k*_–1_	ERC between tissue and blood	34.7	L/day	H
ϕ	volume fraction of exposed Mg in the bone	0.3	-	R
ξ_1_	EqC between exposed and unexposed in serum	0.538	-	R
ξ_2_	EqC between exposed serum and RBCs	0.452	-	R
ξ_3_	EqC between exposed and unexposed in tissue	3.00	-	R

a Under the Source column, V indicates an estimate from various sources^6,7,16–18^ for a 3.2 × 32 mm screw (see Supporting Information B, which will be variable depending on Mg alloy and size (number) of implant(s)), R is derived using the data in Tables S2 and S3 in Supporting Information B, I is derived using Mg isotope tracer data,^12,25,26^ and H is derived from homoeostasis concentration ratios.^13,14^ ERC = exchange rate constant and EqC = equilibrium concentration ratio constant.

#### 2.1.2 Simplified Formulas and Predictions for Long-Term Mg Accumulation

The system of eqs 1–4 can be approximated very well using relatively simple formulas derived on the assumption that the volume of the tissue local to the implant is small compared to the total volume of tissue in the body (i.e., *V*_I_/*V*_T_tot__ = ξ ≪ 1). In Supporting Information C.3, two such formulations are derived, the simplest discussed in Section C.3.1 yields the approximations

6 $$C_{s}^{*}\approx C_{N}^{*}\approx C_{T}^{*}\approx 1+\frac{\sigma \left(1-\xi \right)}{\phi_{D}}\left(1-e^{-t/T_{Mg}}\right)$$

7 $$C_{I}^{*}\approx 1+\frac{\sigma \gamma }{k_{1}\phi_{D}}\frac{V_{T}}{V_{I}}\left(1-e^{-t/T_{1}}\right)+\frac{\sigma \left(1-\xi \right)}{\phi_{D}}\left(1-e^{-t/T_{Mg}}\right)$$

where the formulas for *T*_1_ and *T*_Mg_ are given in Table 2; though 1 – ξ ≈ 1 by these assumptions, the 1 – ξ term is retained to account for the multiple/larger implant cases to be investigated. Here, *T*_1_ is the time point within the time scale for the rapid buildup of Mg in the tissue local to the implant site (about 6 days using values in Table 1), and *T*_Mg_ is the time point within the time scale for the systemic rise in Mg concentration as a result of the biodegrading implant toward the new steady-state concentrations. We note that the “time points” T_*_ is where for a exponentially changing concentration from *C*_old_ to *C*_new_ satisfies (*C*(T_*_) – *C*_old_)/(*C*_new_ – *C*_old_) = 1 – e^–1^ ≈ 0.632, providing a rough indication of the time scale of significant change of the concentration. From [Disp-formula eq6], hypermagnesemia will eventually occur if the Mg release rate from the implant satisfies σ ≳ σ_hyp_ ≈ 1.41 mmol/day in a time scale represented by *T*_hyp_ (formula given in Table 2). When ξ = *V*_I_/*V*_T_tot__ is small, the formulas in Table 2 provide very good quantitative approximations to the numerical simulations of the full system, particularly for *C*_s_, *C*_T_, and *C*_I_. The second formulation presented in Supporting Information C.3 is more rigorous and provides improved agreement with the numerical solutions throughout the implant’s life span (see Supporting Information C.3.2) and is needed in Section 2.2.3; however, the solutions are more complex and less transparent in terms of identifying which mechanisms are most important and when.

**Table 2:** Important Formulae Resulting from the Simplified System Discussed in Section 2.1.2 ^a^

explanation	Formulation
local tissue buildup time point	$T_{1}=\frac{V_{T}\left(1+\xi_{3}\right)}{k_{-1}}\approx 6\;days$
systemic Mg buildup time point	$T_{Mg}=\frac{1}{\gamma }\left(\left(1+\xi_{1}\right)V_{s}+\xi_{2}V_{r}+\frac{\mu_{1}}{\mu_{-1}}\phi V_{N}+\left(1+\xi_{3}\right)\frac{k_{1}}{k_{-1}}V_{T_{tot}}\right)\approx 104\;days$
“critical” implant Mg release rate	$\sigma_{hyp}=\frac{\phi_{D}\left(C_{hyp}^{*}-1\right)}{\left(1-\xi \right)}\approx 1.41\;mmol/day$
time point for hypermagnesemia when σ > σ_hyp_	$T_{hyp}\approx T_{Mg}\;\ln\left(\frac{\sigma }{\sigma -\sigma_{hyp}}\right)$

a The parameter symbols and estimated values are presented in Table 1, with *C*_hyp_^*^ = *C*_hyp_/*C*_hom_.

The predicted compartmental accumulation in the long term are given by the steady-state solutions of ([Disp-formula eq1])-([Disp-formula eq4]), which are

8 $$\left(C_{s_{\infty }}^{*},C_{N_{\infty }}^{*},C_{T_{\infty }}^{*},C_{I_{\infty }}^{*}\right)=\left(1+\frac{\sigma }{\phi_{D}},1+\frac{\sigma }{\phi_{D}},1+\frac{\sigma }{\phi_{D}},1+\frac{\sigma }{\phi_{D}}+\frac{\sigma \gamma }{\xi k_{1}\phi_{D}}\right)$$

in the normalized form; the details are discussed in Supporting Information C.2. These quantities are representative of the compartmental Mg concentrations for a long-term implant (1–2 years). We note that *C*s__∞__, *C*N__∞__, and *C*T__∞__ increase by the same proportion σ/ϕ_D_, depending on the Mg release rate σ and dietary intake rate ϕ_D_. These formulas make the potential maximum Mg concentration explicit in each of the compartments due to the biodegrading implant; this is discussed further in Section 2.3.3.

The approximations [Disp-formula eq6]–[Disp-formula eq8] provide insights into the sensitivity of the parameters on the model predictions. From these equations, the compartmental Mg concentrations increase linearly, at all time points, with an increased Mg release rate σ. The formula for the time point *T*_Mg_ suggest that the time scale for the prolonged gradual rise in Mg linearly increases with increased compartmental volumes and Mg exchange rate ratios, μ_1_/μ_–1_, and *k*_1_/*k*_–1_, and is inversely proportional to the excretion rate γ, while notably independent of the Mg release rate. Moreover, the disparity in volumes between the compartments means that the tissue contributes to around 74% and bone about 25% of this buildup time, using the data in Table 1. The effects of the implant size and/or number (σ, ξ), kidney function (γ), and dietary control are explored below.

#### 2.1.3 Numerical Methods and Validation

Simulations of eqs 1–4 were generated using MATLAB’s differential equation solver ode15s (code available on request). The results presented agree with the mathematically derived approximations presented in Section 2.1.2 and in Section C.3 of the Supporting Information C.

### 2.2 Results

In all time varying simulations of eqs 1–4, the implant is installed at *t* = 0 with the compartmental Mg­(II) ion concentrations being at homeostatic levels given by [Disp-formula eq5]. The “standard simulation” involves Mg release from a single 3.2 × 32 mm pin/screw at a rate of σ = 0.05 mmol/day, with all other parameters given in Table 1. Further simulations will investigate outcome of multiple Mg screw implants (Section 2.2.2), compromised kidney function, and dietary control (Section 2.2.3). The multiple implant simulations also reflect results for larger Mg implant devices with an equivalent surface area. Using the information in Supporting Information B, a normalized concentration of *C*_hyp_^*^ = *C*_hyp_/*C*_hom_ ≈ 1.24 is the hypermagnesemia threshold and *C*_sev_^*^ = *C*_sev_/*C*_hom_ ≈ 3.41 is considered a dangerous level.

#### 2.2.1 Effect of Corrosion of a Single Mg Screw Implant

Figure 2 shows the evolution of the normalized exposed Mg­(II) ion concentration, *C*_j_^*^, for a single implant case. The concentration of Mg local to the implant, *C*_I_, rises relatively rapidly for the first 2–3 weeks, while *C*_s_, *C*_N_, *C*_T_ remain relatively unchanged (note the normalized concentrations overlap). The Mg concentration in the implant region reaches beyond that of hypermagnesemia (*C*_I_^*^ ≈ 1.24) and “dangerous” levels (*C*_I_^*^ ≈ 3.41), while that of rest of the compartments eventually peaks at around 0.8% above homeostatic levels. The Mg concentration in the implant region reaches the steady, elevated level from around 3 weeks, broadly agreeing with the experimental observation.^29^ The dotted line labeled indicates the time point *t* = *T*_Mg_ (see Table 2). The results here suggests that for a healthy individual, a single, small Mg based implant will make little impact on systemic Mg concentrations, which is in broad agreement with a number of magnesium implant studies.^7,16,17^

**Figure 2:**
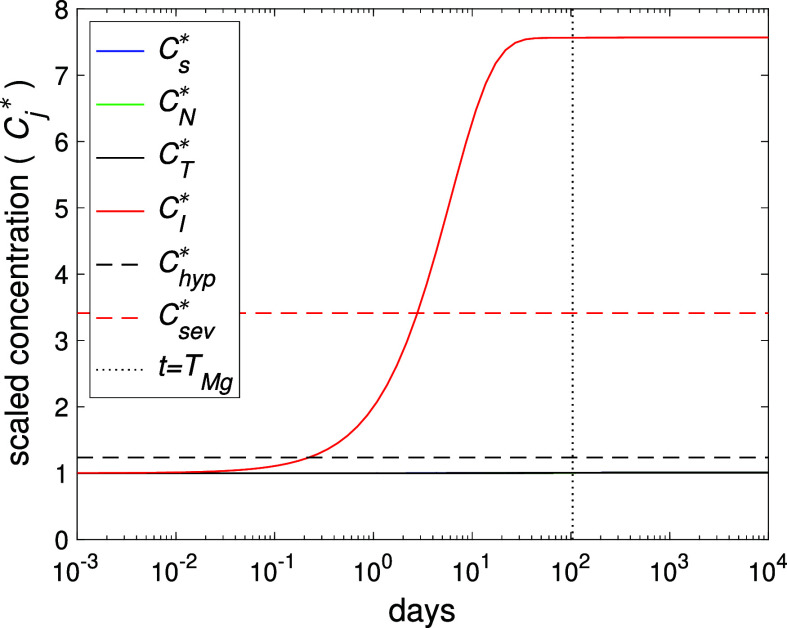
Plot of the normalized concentrations $\left(C_{j}^{*}=C_{j}/{\mathrm{C̅}}_{j}^{e}\right)$ over time, resulting from eqs 1–4 using the parameter values in Table 1 and σ = 0.05 mmol/day. The dotted line indicates *t* = *T*_Mg_ ≈ 104 days from Table 2 and discussed in Section 2.1.2. The variables *C*_s_^*^, *C*_N_^*^, *C*_T_^*^ all overlap each other.

#### 2.2.2 Effect of Multiple or Larger Implants

For severe bone trauma, multiple pins and screws may be necessary to fix the bones. Figure 3 shows the evolution of the normalized concentrations *C*_s_^*^ (Figure 3A) and *C*_I_^*^ (Figure 3B) in time for a range of multiple implants. Here, we assumed that the regions of tissue immediately effected by each implant is so localized that they do not overlap each other (though overlapping will not significantly affect the results), which means the following parameter modifications to describe *n* implants are made

9 $$V_{I}=nV_{I_{0}},,\sigma =n\sigma_{0},,\xi =n\xi_{0}$$

where *V*_I_0__, σ_0_, and ξ_0_ are the corresponding values for one implant (*n* = 1), noting that *V*_I_ < *V*_T_tot__ (ξ < 1) is required for no overlap of the implant zones. The diffusion distance of elevated Mg in tissue is observed to be 3–4 mm,^29^ so the no overlap assumption directly applies to multiple screws spaced more than 6–8 mm apart. Note that [Disp-formula eq9] could also represent a single larger or porous implant that has *n* times the surface area of a typical screw implant.

**Figure 3:**
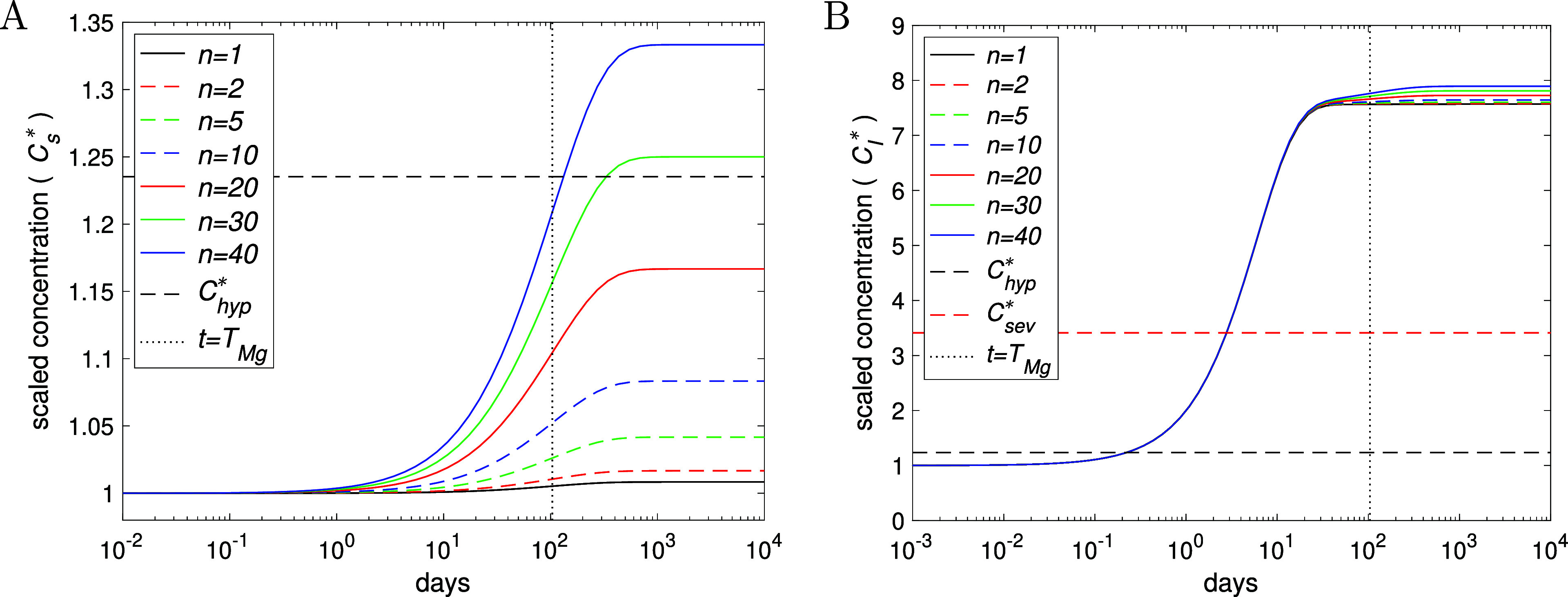
Plots of the normalized concentrations (A) *C*_s_^*^ (noting *C*_T_^*^ and *C*_N_^*^ are visually identical) and (B) *C*_I_^*^ concentrations over time for the indicated number of implant devices *n*, resulting from eqs 1–4. The black and red dashed lines show the hypermagnesemia threshold (*C*_hyp_^*^ ≈ 1.24) and approximate “dangerous” symptomatic threshold (*C*_sev_^*^ ≈ 3.41, plot (B) only), respectively; note the hypermagnesemia threshold is exceeded when *n* ≥ 29 (*n* = 29 case is the dot-dashed curve). The vertical dots indicate *t* = *T*_Mg_ ≈ 104 days from Table 2. All parameters are listed in Table 1, with *V*_I_, σ, ξ changed according to 9.

As expected, increasing *n* increases the Mg concentration in each of the compartments. The simulations suggest that a fairly considerable number of implants is required to lift serum levels of Mg to a state of hypermagnesemia in a healthy individual (see Figure 3A), even without employing an active adaptation process to regulate Mg levels (these results can thus be considered a worst-case scenario); here, the minimum number of implants leading to hypermagnesemia is σ_hyp_/σ_0_ ≈ 28–29 from Table 2 and 9. Initially, *C*_I_^*^ increases at around the same rate for the first 2–3 weeks, independent of the number of implants, as there is no overlap of the zones of locally effected tissue (see Figure 3B). As expected, more implants mean a more rapid rise of Mg in serum, seemingly starting from about 2 days. However, the time scale to reach close to the steady-state concentration, given by [Disp-formula eq8], is about 2–3 years in all cases, indicating that systemic Mg accumulation is a process occurring on a long time scale (longer than the lifespan of a typical Mg based implant^1^). The profiles of *C*_N_^*^ and *C*_T_^*^ are similar to that shown for *C*_s_^*^, though the increase of Mg in bone initially lags behind (as illustrated in Figure 4). The long time scale for Mg to settle in the body is due to the relatively large volumes of bone and tissue and their high Mg load, for which a considerable amount of Mg needs to be released and absorbed before it impacts on their concentration; consequently, the model predicts that tissue and bones play a key part in regulating the long-term serum Mg concentration. This long time scale of Mg accumulation was observed in reports by Zhang et al.,^30^ whereby daily Mg supplements were taken orally (0.4 mmol/day, roughly an additional 10% of normal daily intake) by healthy subjects and measurements made using urine samples. They observed that supplements had the effect of raising the Mg content in the urine, reaching a plateau around 6 months.

**Figure 4:**
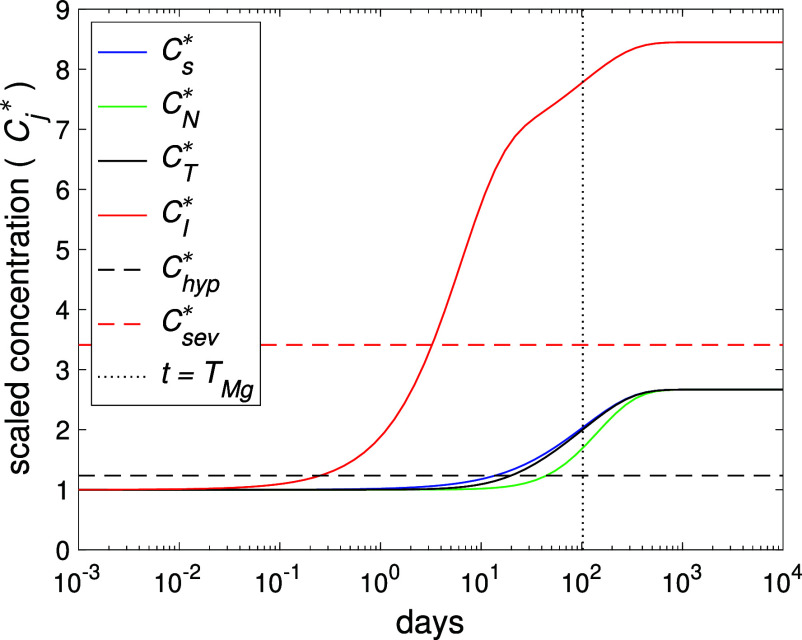
Plot of the normalized concentrations *C*_j_^*^ over time, resulting from eqs 1–4, for the scaled up Sato et al.^17^ experiment, involving a Mg plate with approximate dimension 424 × 141 × 1 mm and release rate of σ = 10 mmol/day. The black dashed line indicates the hypermagnesemia threshold, *C*_hyp_^*^ ≈ 1.24 and the red dashed line indicates the “problematic level”, *C*_sev_^*^ ≈ 3.41. The vertical dots indicate *t* = *T*_Mg_ ≈ 104 days given from Table 2. All parameters are listed in Table 1, with ξ ≈ 0.023 corresponding to the size of the plate.

The *in vivo* experiments of Sato et al.^17^ involved inserting a 30 × 10 × 1 mm Mg alloy plate in the subcutaneous layer on the back of a rat for 55 h. Scaled up to human proportions (about 200 times in volume), a plate with the same aspect ratio and 1 mm thick would have dimensions around 424 × 141 × 1 mm (see Supporting Information B). Figure 4 shows the Mg concentration profiles for the scaled up case as predicted by the model (using *V*_I_ = 20× the volume of the plate, representing about 1.2 L of tissue). In reality, an implant of such dimension, with the associated tissue damage on instalment, is likely to create a clinical scenario that is beyond the intended scope of the model; however, the Mg release rate may be reflective of smaller porous implants with a higher surface to volume ratio, for example, Mg wire scaffolds for use in bone tumors.^31^ The model simulations show that the implant has little effect on serum and tissue Mg concentrations in the first few days (up to around *t* = 5 – 10 days), in agreement with the experimental results. However, the results underestimate the recorded Mg excretion rate and the rate of Mg increase in localized tissue concentration; though quantitative agreement for the latter can be tuned by decreasing the volume *V*_I_.

#### 2.2.3 Effect of Reduced Kidney Function and Dietary Mg Intake Control

A well-functioning kidney is usually sufficient at preventing hypermagnesemia due to being able to increase excretion rate in response to elevated levels. A measure of health of the kidney is the GFR (glomerular filtration rate), whereby healthy young adults typically have a GFR of 100+ mL/min/1.73 m^2^ that declines steadily with age to around 70 mL/min/1.73 m^2^ in the elderly;^32^ though these figures vary with gender and race. Typically, hypermagnesemia is only experienced by patients with a compromised renal function due to chronic renal failure, often with Mg containing medications, when GFR falls below 30 mL/min/1.73 m^2^,^14^ for which the serum Mg concentration is monitored and intake carefully managed. For the simulations until now, the urinary excretion parameter (γ) was kept constant for the well-functioning kidney case, that is, γ = γ_0_, where γ_0_ is the value of γ in Table 1. Reduced kidney function corresponds to γ < γ_0_ with a corresponding reduction in the Mg intake rate ϕ_D_ so the Mg concentration is at homeostasis, that is, at $C_{s}=\phi_{D}/\gamma \approx {\mathrm{C̅}}_{s}^{e}$ , prior to implant instalment. Figure 5 shows the effect of the dimensionless parameter Γ = γ/*k*_1_ on the steady-state *C*_s_^*^ concentration (Figure 5A, using the formulas in [Disp-formula eq8]) and concentration at one year after the introduction of the implant (Figure 5B, using [Disp-formula eq6] evaluated at *t* = 365 days) for a various number of implants; note that γ = γ_0_ corresponds to Γ = Γ_0_ ≈ 0.0787. Though dependent on the type of Mg alloy and size of implant, a typical lifespan of the material is around one year,^6,7^ so Figure 5B represents a more realistic estimate of the maximum serum Mg concentration relative to the homeostatic level. In both plots, the vertical black dots indicate the healthy Mg turnover rate, while the vertical red dots indicate roughly the chronic renal failure threshold (i.e., a GFR of about a third of the healthy level); we note that patients with a GFR corresponding to the left of the red dotted line will most likely be too sick to receive an implant procedure. The figures demonstrate that serum Mg concentration decreases with Γ and increases with implant number *n*, as to be expected, though the model predicts that a large number of implants (*n* = 10+ ) are required to drive levels above the hypermagnesemia threshold (when Γ > Γ_0_/3), though this number could be somewhat fewer if the patient is additionally receiving Mg containing medications. The results therefore suggest that the risk of hypermagnesemia is extremely low in patients with a reduced GFR from a small number of screw/pin implants. Figure 6 shows the hypermagnesemia onset time *T*_hyp_, using the formula in Table 2, as a function of the implant(s) Mg release rate, σ, equivalent from 0 to 100 screw implants. As expected, for a given release rate, the onset of hypermagnesemia will occur sooner for cases with compromised kidney function; however, even for a large number or size of implants, it could take several months to occur.

**Figure 5:**
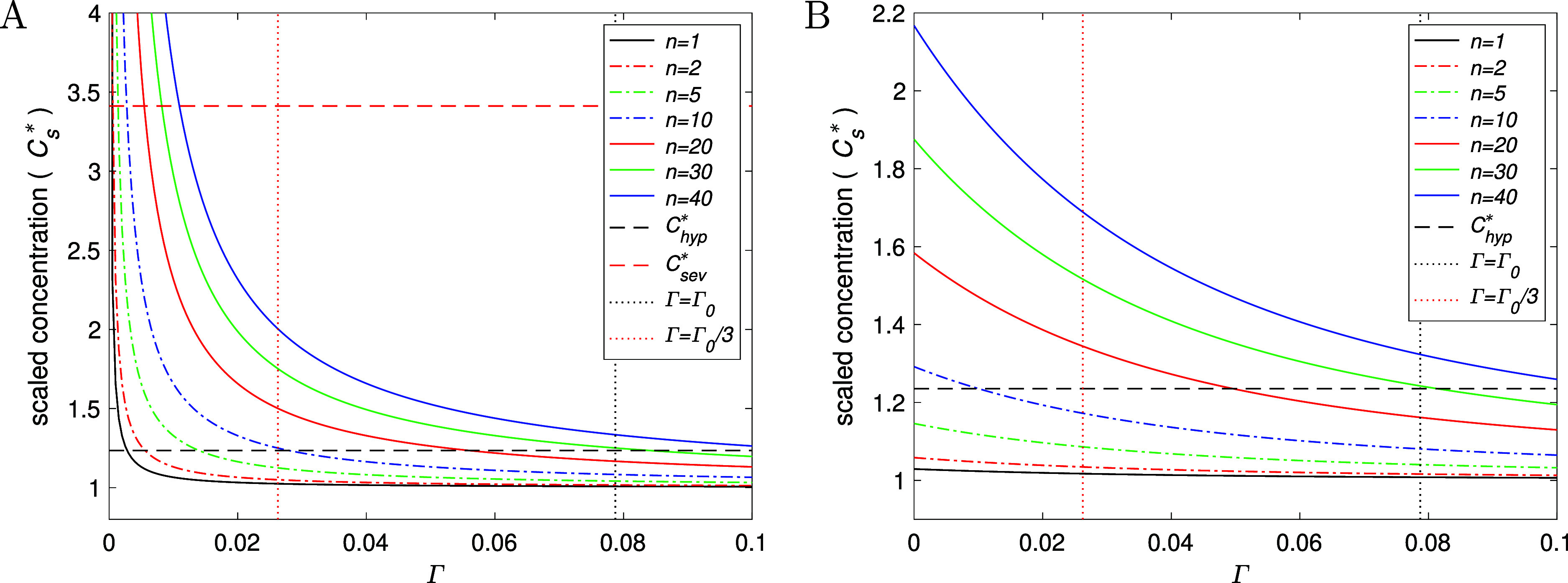
Plots of normalized serum concentration of Mg at steady-state $\left(C_{s_{\infty }}^{*}\right)$ using [Disp-formula eq8] (plot A) and at 12 months (B) using [Disp-formula eq6] against Γ = γ/*k*_1_ for a different number of implants *n*. Hypermagnesemia corresponds to *C*_hyp_^*^ ≈ 1.24 and “problematic level” to *C*_sev_^*^ ≈ 3.41. All other parameters given in Table 1, with *V*_I_, σ, ξ changed according to 9.

**Figure 6:**
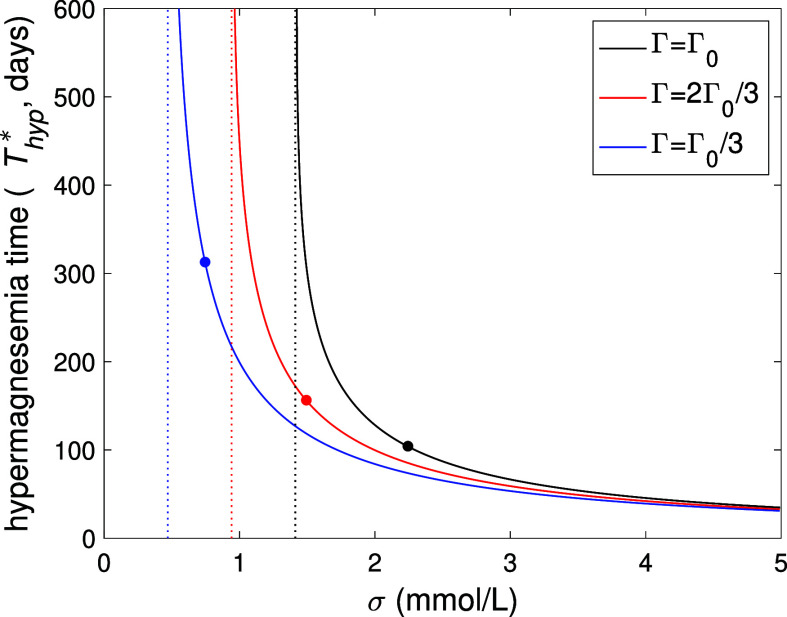
Plots of the onset time for hypermagnesemia (*T*_hyp_ when *C*_s_^*^(*T*_hyp_) = *C*_hyp_^*^ ≈ 1.24), using the formula in Table 2, against the implant Mg release rate (σ, with σ = 0.05 being equivalent to a single screw implant) for various levels of kidney function characterized by Γ = Γ_0_, 2Γ_0_/3 and Γ_0_/3. The vertical dotted line indicate the minimum release rate for hypermagnesemia in each case, and the dots correspond to *C*_s_^*^(*T*_Mg_) = *C*_hyp_^*^. All other parameters given in Table 1.

Patients at risk of hypermagnesemia, via kidney malfunction, etc., will have their Mg levels monitored and intake controlled through drugs (including drug withdrawal) and diet; for convenience, we will refer to these as external sources of Mg. From the modeling point of view, the implant represents an additional (internal) form of Mg intake, which cannot itself be controlled easily once installed; hence, any clinical control can only be achieved via regulating the external Mg sources. To model this, we introduce the parameter ρ, such that ρ = 1 represents normal Mg intake and ρ = 0 is zero intake from external sources, which leads to the following modification of eq 1

10 $$\left(V_{s}\left(1+\xi_{1}\right)+V_{r}\xi_{2}\right)\frac{dC_{s}}{dt}=\rho \phi_{D}-\left(\gamma +\mu_{1}+k_{1}\right)C_{s}+\mu_{-1}C_{N}+k_{-1}\left(\left(1-\xi \right)C_{T}+\xi C_{I}\right)$$

Figure 7 shows the effect of reducing the external Mg intake (reduction starting at *t* = 0 for a patient with severely compromised kidney function (γ = γ_0_/3) with, for illustrative purposes, an equivalent Mg release rate of *n* = 20 small screw implants. As expected, reducing the intake reduces the long-term serum Mg concentration, whereby a reduction to ρ = 0.5 (halving the dietary intake) leads to *C*_s_ being close to homeostatic levels throughout (see Figure 7A), with a small dip in the earlier phases before rising around *t* ≈ *T*_Mg_ (the formula of which is unchanged from that in Table 2, see Supporting Information C.3.1.3). Figure 7B shows that variation in dietary intake has relatively little effect on the Mg concentration in the implant region. The steady-states of eqs 2–4 and 10 are given by

11 $$\left(C_{s_{\infty }}^{*},C_{N_{\infty }}^{*},C_{T_{\infty }}^{*},C_{I_{\infty }}^{*}\right)=\left(\rho +\frac{\sigma }{\phi_{D}},\rho +\frac{\sigma }{\phi_{D}},\rho +\frac{\sigma }{\phi_{D}},\rho +\frac{\sigma }{\phi_{D}}+\frac{\sigma \gamma }{\xi k_{1}\phi_{D}}\right)$$

providing an estimate of ρ to guarantee avoidance of hypermagnesemia; for example, to maintain homeostasis levels in the long term (i.e., C_s_∞__ ≈ 1) we need

12 $$\rho \approx 1-\frac{\sigma }{\phi_{D}}$$

**Figure 7:**
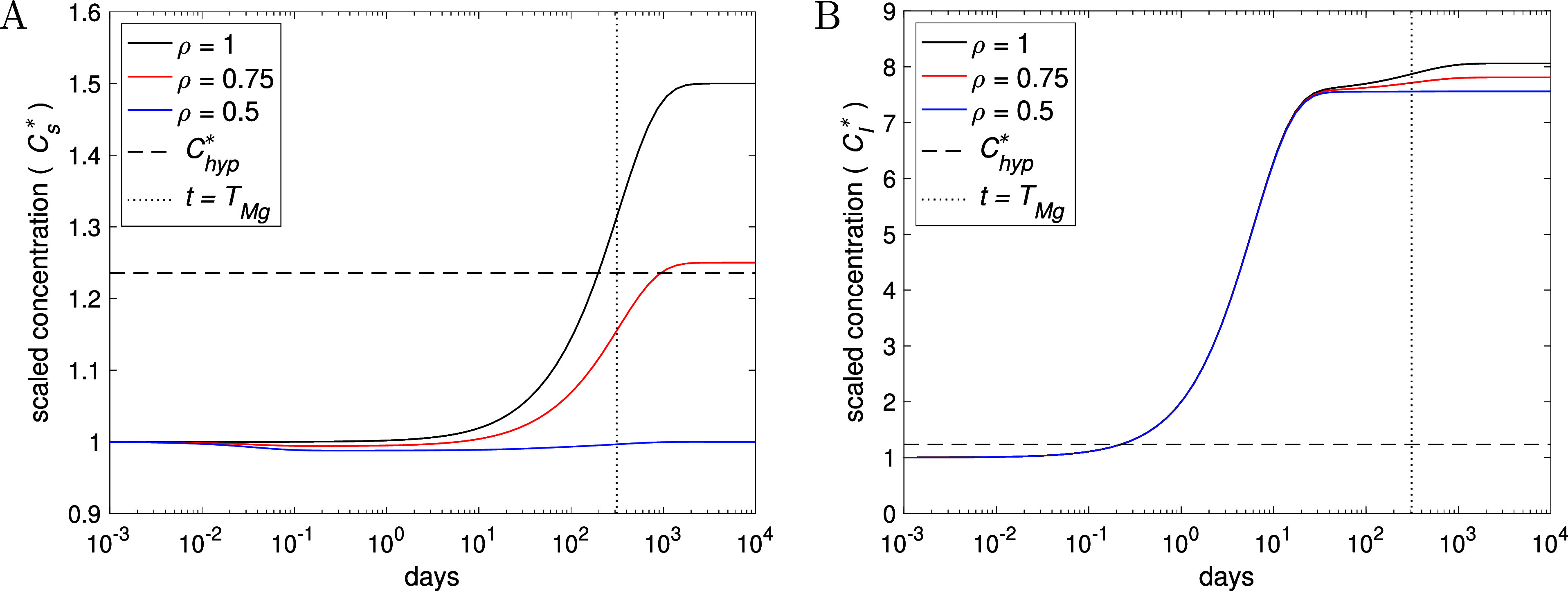
Plots showing the effect of Mg intake control (ρ) on Mg serum (A) and localized tissue (B) concentration for an individual with a severely defective kidney (γ = γ_0_/3) and *n* = 20 implants, resulting from eqs 2–4 and 10. The dotted line indicate *t* = *T*_Mg_ ≈ 104 days from Table 2. All other parameters given in Table 1, with *V*_I_, σ, and ξ changed according to 9.

The reduction in Mg intake may perhaps lead to hypomagnesemia (i.e., when *C*_s_^*^ ≲ 0.76–0.88, equivalent to 0.65–0.75 mmol/mL^10,11^ ), indeed we observe a small drop in serum Mg concentration in Figure 7A around 1–10 days. From Supporting Information C.3.2, it is shown that minimum Mg concentration is $C_{s_{\min}}^{*}\approx 1+\left(\rho -1\right)/\gamma k_{1}$ , whereby using the parameters in Table 1, the maximum drop is predicted to be about 8% of homeostatic levels when Mg intake is zero (i.e., ρ = 0), comfortably above the level for hypomagnesemia; here, the model predicts Mg levels in serum to be largely maintained up to around *t* = *T*_Mg_ by the Mg reserves in tissue and bone compartments.

### 2.3 Discussion

The proposed PBPK model was developed to predict the changes in time of Mg concentration in blood, bone, and tissues throughout the lifetime of one or more biodegradable Mg bone implants. The Mg­(II) ions released by the implant are carried in the blood, where they diffuse and accumulate in the tissue and bone as well as being excreted via the gut and kidneys. The PBPK model enables predictions to be made about the ultimate outcome of these processes over a long time period. The current model incorporates a passive adaptation process via paracellular transport^15^ in the gut and kidney to regulate Mg levels, and the presented results represent a worst-case scenario in healthy individuals and are most relevant to individuals with compromised kidney function.

#### 2.3.1 Model Predictions of Systemic Mg Levels for Short and Long-Term

For the first 3 weeks or so, the model predicts that the Mg concentration local to the implant, *C*_I_, rises relatively rapidly (around the time point *T*_1_ ≈ 6 days) to a temporary, elevated saturated level (in agreement of Zhang et al.^29^). The concentration in the serum, bone, and tissue compartments (*C*_s_, *C*_N_, *C*_T_) remains at homeostaic levels, in broad agreement with published results.^17,29^ The model further predicts that the localized tissues will be exposed to this elevated Mg concentration, possibly well beyond that for clinical hypermagnesemia, throughout the implant’s life span;^29^ we note that the saturated localized concentration is related to the inverse of implant-zone tissue volume *V*_I_ (see eq 7), and since *V*_I_ cannot be precisely defined from experiments, data on *C*_I_ can be used to provide an estimate of *V*_I_ for different scenarios. Regardless of this uncertainty, the volume represents only a small fraction of the total tissue volume and the longer-term predictions of *C*_s_, *C*_N_, *C*_T_ are relatively unaffected by the parameter *V*_I_.

The key model prediction is that it will take several months before Mg levels are observed to increase in the blood, taking nearly two years to settle to the steady, maximal levels, consistent with experimental observations.^30^ From the simplified formulation of Section 2.1.2, built on the assumption that *V*_I_/*V*_T_tot__ is very small, a representative time scale for this systemic rise is indicated by the time point *T*_Mg_ ≈ 104 days. Although this value changes with the excretion rate or intake rate via relation $\phi_{D}/\gamma ={\mathrm{C̅}}_{s}^{e}$ at homeostasis, the time scale for systemic increase is several months in a clinically relevant setting (see for example Figure 6). However, for a small number and/or size of implants, such a systemic rise in Mg concentration is unlikely to be noticeable against day-to-day variations, but this may not be the case if significantly more and/or larger implants are installed.

#### 2.3.2 Number and/or Size of the Implants and Dietary Control

A typical implant for humans (e.g., a screw, say of diameter 3.2 mm and length 32 mm) is estimated to degrade at around σ = 0.05 mmol/day,^6,7,16,17^ which is considerably less than that absorbed via the intestine (about ϕ_D_ = 4–6 mmol/day). We note this release rate will be variable depending on the Mg alloy, location, patient’s size, and health circumstances (e.g., with or without osteoporosis, compromised kidney function). Nevertheless, the maximum extent to which such an implant will increase the daily uptake of Mg will be no more than 2–3%, well short of the 100*C*_hyp_/*C*_hom_ = 24% or so required for hypermagnesemia, where *C*_hom_ and *C*_hyp_ are the homeostatic and hypermagnesemia threshold serum Mg concentrations, respectively. The model predicts that the long-term systemic Mg concentration increases linearly with the implant Mg release rate σ, representing an increase in number (via [Disp-formula eq9]) and/or the size of the implants. Using the steady-state formulations [Disp-formula eq8], the maximum percentage increase in systemic Mg is predicted to be 100σ/ϕ_D_, with hypermagnesemia being possible if σ ≳ ϕ_D_(*C*_hyp_ – *C*_hom_)/*C*_hom_. Using the data in Table 1, this translates to around *n* = 29 screws of dimension 3.2 × 32 mm (or equivalently a single 1 × 10 cm rod) for a patient with healthy kidneys (corresponding to the γ value in Table 1). However, the number/size of implants reduces significantly on decreased kidney function (see Figure 5), for example, down to *n* = 10–15 screws for a GFR about one-third of the healthy level. Furthermore, Figure 6 shows that the time taken to reach hypermagnesemia, *T*_hyp_, decreases with the number/size of implants as well as with kidney function, and most notably, it will take several months before hypermagnesemia is observed in clinically relevant cases. We are not aware of any human studies to directly validate these results, but Zheng et al.^18^ recently investigated the long-term effects of Mg implants on a rat model for chronic kidney disease. Their results showed an elevation of around 20% in serum levels after 12 weeks for the pure Mg implant that is broadly in line with the model predictions. They also acknowledged, however, the limitation of their animal model in terms of representing the stages of chronic kidney disease in humans. We stress once again that these results are most relevant for the cases of compromised kidney function, as the current model does not account for active Mg regulation by the kidney. Though the stated numbers and/or dimension of implants may only be relevant for the most severe bone repair procedures, they may be reasonable in other applications of Mg implants, such as H_2_ emitting devices for controlling tumor growth^33^ and the potential use of Mg wire scaffolds for bone tumors that provides mechanical support for the defective bone and suppress tumor growth.^31,34,35^

For patients with severe relevant health concerns, management of Mg in the diet can provide an effective means of controlling Mg levels in the body; this is represented in the model by tuning parameter ρ according to 12 to maintain homeostasis. The model predicts that a reduction in Mg intake will lower systemic levels, but there is little risk of hypomagnesemia. If the circumstances are such that [Disp-formula eq12] gives a negative value for ρ, then a practical alternative would be to set ρ < *C*_hyp_^*^ – σ/ϕ_D_ to at least ensure that hypermagnesemia is avoided. These predictions are based on a constant controlled diet with an implant releasing Mg at a constant rate. In reality, there could be fluctuations in patient’s health and in the Mg release rate of the degrading implant, meaning that a fixed dietary prescription of Mg intake will not always be applicable. Given the model prediction of systemic Mg accumulation being a long-term process, the results suggest that Mg levels should be monitored in vulnerable patients throughout the lifetime of the implant and Mg intake should be controlled accordingly.

#### 2.3.3 Model Applicability and Limitations

A pleasing attribute of the model is that many of its parameters can be estimated from the experimental literature. Intake rate, excretion rate, GFR, homeostatic Mg blood serum concentration, and bodily volumes are readily measurable quantities; taking gender, size, age, circumstance, and race into account, good progress can be made toward tailoring the model to simulate long-time outcomes of Mg implants for patient specific cases. Of course, there will be further variation depending on the alloy type, size, location of the Mg implant, and dietary intake of Mg; however, the formulas presented in Section 2.1.2 provide a simple means of predicting how such variations could effect the long-term accumulation of Mg in the body. For example, we can see from the formula for *T*_Mg_ (Table 2) and eq 6 that the time taken for a noticeable rise in systemic Mg is increased in larger individuals (via increased *V*_N_ and *V*_T_tot__). There appears to be no published data that enable direct estimation of the Mg mass transfer rates *k*_1_, *k*_–1_, μ and μ_–1_. As described in Supporting Information B, the ratios μ_1_/μ_–1_ and *k*_1_/*k*_–1_ are straightforward to establish from published data and by reproducing the published results^26^ provides a further means of estimation. However, a degree of freedom remains, for which we used the generic pharmacological data to estimate the ratio *k*_1_/μ_1_;^36^ any experiments that can address this degree of freedom for Mg would be useful to completely parametrize the model. Nevertheless, knowledge of the ratios μ_1_/μ_–1_ and *k*_1_/*k*_–1_ are sufficient for the model to numerically predict the long-term compartmental concentrations and the time scale *T*_Mg_ and *T*_hyp_, from which the main conclusions of this paper are drawn. A further attribute of the model is that it is amenable to mathematical analysis in the clinically relevant case of *V*_I_/*V*_T_tot__ ≪ 1, enabling the derivation of simple formulas for the compartmental Mg concentrations as functions of time, key time scales, and critical Mg release rates (or equivalently the critical number and/or size of implant) that could potentially lead to hypermagnesemia in the long term (see Table 2); such simple formulas, once validated, could be useful to inform clinical decisions for vulnerable patients.

The main weakness of the current model is that it does not take into account the any “active” regulatory processes controlling Mg levels in the body via excretion/resorption in kidneys, gut, and with bone;^13^ only passive responses (by paracellular transport^15^) are considered via assuming a linear rates for excretion and Mg exchange between serum and bone. These processes are complex and add extra layers of interaction between chemical species (calcium, phosphates, sodium, etc.) and hormonal regulation; however, a good deal is known about Mg physiology and kinetics and can be incorporated in an extended model. Furthermore, we only considered implants with a constant release rate of Mg, this being consistent with experimental observation;^6^ but in other situations, this could be variable due to different coatings,^37^ alloys, geometry, formation of corrosion layers (e.g., calcium phosphate^38^), and surface pitting; for this, we can make σ time-dependent, or couple σ with a dynamic equation for implant mass. The assumption of constant bone mass during the lifespan of the implant could also be challenged, for example, Mg ions was shown to enhance bone mass of rats with osteoporosis;^39^ however, this will not change the key results unless the change in bone mass is significant. A further refinement of the model is to include more compartments representing, for example, a greater range of soft tissues (muscle, skin, liver, etc.) that could, if necessary, provide greater quantitative predictability. Such additions will of course complicate the model, but initial estimates for the new parameters are available in pharmacology texts^36^ and the additional ODEs will largely be linear, so the mathematical approaches used to derive the simplified formulas can be carried forward to a more detailed model.

The current model demonstrates that it is possible to describe quantitatively the long-term fate of a magnesium implant based on the well-established principles of pharmacokinetics. It forms a basis that can be adapted to represent various clinical situations, accounting for patient age and body mass, renal function, health status, etc. Though PBPK models have been around for some time, this is the first time, to our knowledge, that this has been applied to describe systematically the Mg distribution resulting from the degradation of Mg implants. It is worth noting that the modeling is generic and is likely to be applicable to many other degradable implant materials, such as polymers^40^ and zinc^41^ implants. To our knowledge, there has been no long-term monitoring over 2–12 months of Mg levels in human studies reported in the literature, while recent progress is being made using animal models;^18^ such data would be invaluable for model validation and continued development. A well-developed and validated model could therefore be used to predict implant performance and aid manufacturers to establish product specification, satisfying the regulatory requirements in terms of risk control via postimplantation monitoring and postmarket surveillance.^42^

## 3 Conclusion

This is the first time, to our knowledge, that a PBPK modeling approach has been applied to predict the long-term, systemic Mg concentration distribution resulting from the degradation of Mg implants. Due to the vast volume disparities between bones, tissues, and the vasculature, it is predicted that a significant amount of Mg must be released and absorbed over time, typically several months, before it causes observable changes in tissue and blood concentrations. Consequently, for patients with compromised kidney function, particularly with large or multiple implants, postimplantation monitoring of blood Mg and/or Mg intake control should be undertaken throughout the life span of the implant in order to mitigate against any adverse effects of continued high Mg presence. Through continued development of the modeling framework and validation, the model can be adapted to describe a broad range of biodegradable implant materials, in various clinical situations, accounting for patient age and body mass, renal function, health status, etc., which can inform on implant manufacture and design and decision making in a clinical setting.

## Supplementary Material



## Notes

Supporting information A: Presentation of the full mathematical model and a description of its systematic reduction to the simpler model disscussed in Section 2.1.1; Suppporting information B: Discussion on the estimation of the parameter values presented in Table 1 and used in the simulations of the Results section; Supporting information C: Presentation of the mathematical analysis of the reduced model presented in Section 2, to derive time dependent and long-time formulas for the magnesium ion species (PDF)
