# Supplementary material for: Physiologically Based Pharmacokinetic Model of Magnesium Implant Absorption and Distribution in Tissue and Organs
Source: ACS Omega. 2026 Jan 22;11(4):5144–53. doi: 10.1021/acsomega.5c06910 (PMC12878740; doi:10.1021/acsomega.5c06910)
Supplement: Supplementary file 1 [file ao5c06910_si_001.pdf]

Physiologically based pharmacokinetic model of magnesium implant absorption and distribution in tissue and organs

John P. Ward,*,† Safia K. Ahmed,*,† and Yang Liu*,‡

†*Department of Mathematical Sciences, Loughborough University, Loughborough, U.K.*

‡*Centre of Biological Engineering, Wolfson School of Mechanical, Electrical and Manufacturing Engineering,*

Loughborough University, Loughborough, U.K.

E-mail: john.ward@lboro.ac.uk; safia020@gmail.com; Y.Liu3@lboro.ac.uk

Contents

Supplementary Information A: Model formulation S2

Supplementary Information B: Parameterization S9

Supplementary Information C: Mathematical analysis S16

References S32

Supplementary information A: Model formulation

The model is aimed to described the changes in bodily Mg concentration over time, in response to an Mg implant introduced at $t = 0$. The body is split up into four main compartments, namely bone (quantities with subscript N), tissue (subscript T, consists of muscle and soft-tissues bundled together, see Supplementary information B), blood (separated into plasma, subscript s, and red blood cells (RBCs), subscript r, compartments[1,2]) and, in the presence of an implant, "tissues local to the implant site" (subscript I). Within each compartment the Mg may transferable (or "exposed", superscript e) between compartments (mainly Mg(II) ions) or non-transferable (or "unexposed", superscript u); the details are discussed below. The concentrations of Mg in each of the compartments are denoted C_j^* for $j = \{s, r, N, T, I\}$ and $* = \{e, u\}$. The assumed Mg transportation pathways within the body that is representative of , from which the model is formulated, is shown in Fig. S1.

The essential source of Mg in the blood is from the diet, which, for simplicity, is assumed to be sourced at a constant rate ϕ_D; we will not include in our model adaptation of Mg absorption from the gut[3], however, this parameter will be set appropriately in simulations for the compromised kidney function case. The release rate of magnesium from the implant, σ, is assumed constant (though a time dependent σ can easily be introduced). The constant degradation rate is consistent with the relation $dV/dt \propto -S$, where V is volume and S is surface area of the implant, which can easily be verified for cubes, cylinders, spheroids etc. Moreover, this is experimentally supported from time-course data using CT analysis of a Mg implant in a rabbit[4]. Of course, σ will be dependent on the alloy material, size and number of implants. The rate of Mg release used as a basis in the simulations is for an Mg screw/pin of size 3.2×32 mm, which is a typical dimension for use in fixing bones in humans. Excretion, predominantly via the kidneys[1], is modelled as a Mg(II) ion sink proportional to the exposed Mg blood serum concentration, with rate constant γ, representing a paracellular transport process[3]; again no active adaptation is assumed here[3], other than Mg(II) ions in the urine increasing in proportion to that of the serum levels (reflected by γ being constant).

Table S1: Values for additional model parameters (the rest listed in Table 1) contained in the "full model" for healthy individuals discussed in Supplementary information B. Under the Source column, R is derived using the data in Tables S2 and Table S3 in Supplementary information B, and in the value column, A is unknown but is assumed large in comparison to other dimensionally equivalent parameters.

Parameter	Description	Value	Units	Source
$\overline{C}_s^u$	Homoeostatic unexposed concentration in serum	0.298	mmol/L	R
$\overline{C}_r^u$	Homoeostatic unexposed concentration in RBCs	2.25	mmol/L	R
$\overline{C}_N^u$	Homoeostatic unexposed concentration in bone	43.1	mmol/L	R
$\overline{C}_T^u$	Homoeostatic unexposed concentration in tissue	6.59	mmol/L	R
λ_1	ERC between exposed and unexposed in serum	A	L/day	-
λ_2	ERC between exposed in serum and in RBCs	A	L/day	-
λ_3	ERC between exposed and unexposed in tissue	A	L/day	-

Constants $\phi_D > 0$ and $\gamma > 0$ govern the homoeostatic Mg levels, whereby a compromised kidney (reduced γ) must be balanced with Mg intake so that the ratio ϕ_D/γ is set at a healthy level.

Around 99% of the total magnesium in the human body is stored in the bone and tissue[1], and from there the Mg(II) ion is exchanged to and from the blood compartment. The concentration of Mg in the serum can be assorted into three groups, ionised, protein bound and complexed with anions[1,2]. Of the three assortments, the ionised concentration of Mg in the serum, which represents approximately 65%, is transferable across the compartments and blood serum[1,2]. Within the serum, there is an exchange of Mg between exposed and unexposed compartments, with partitioning and exchange rate constant ξ_1 and λ_1, respectively. The majority of Mg in the RBCs are locked in complexed form, the small amount of ionised Mg(II)[5,6] can be transported between the cell and serum, but never converted to the complexed form within the RBC. The exchange between the exposed form of Mg in cells and serum are characterised by partitioning constant ξ_2 and exchange rate constant λ_2. About 30% (fraction ϕ) of the bone compartment acts as a Mg reservoir and is exchangeable[2] with, what is assumed to be, the vasculature (with exchange rate constants μ_1 and μ_{-1}). In the "unexposed" bone region (fraction $1-\phi$), the Mg levels are assumed to remain unchanged over the timescale of interest ($< 1-2$ years) and there is no transfer of Mg between the

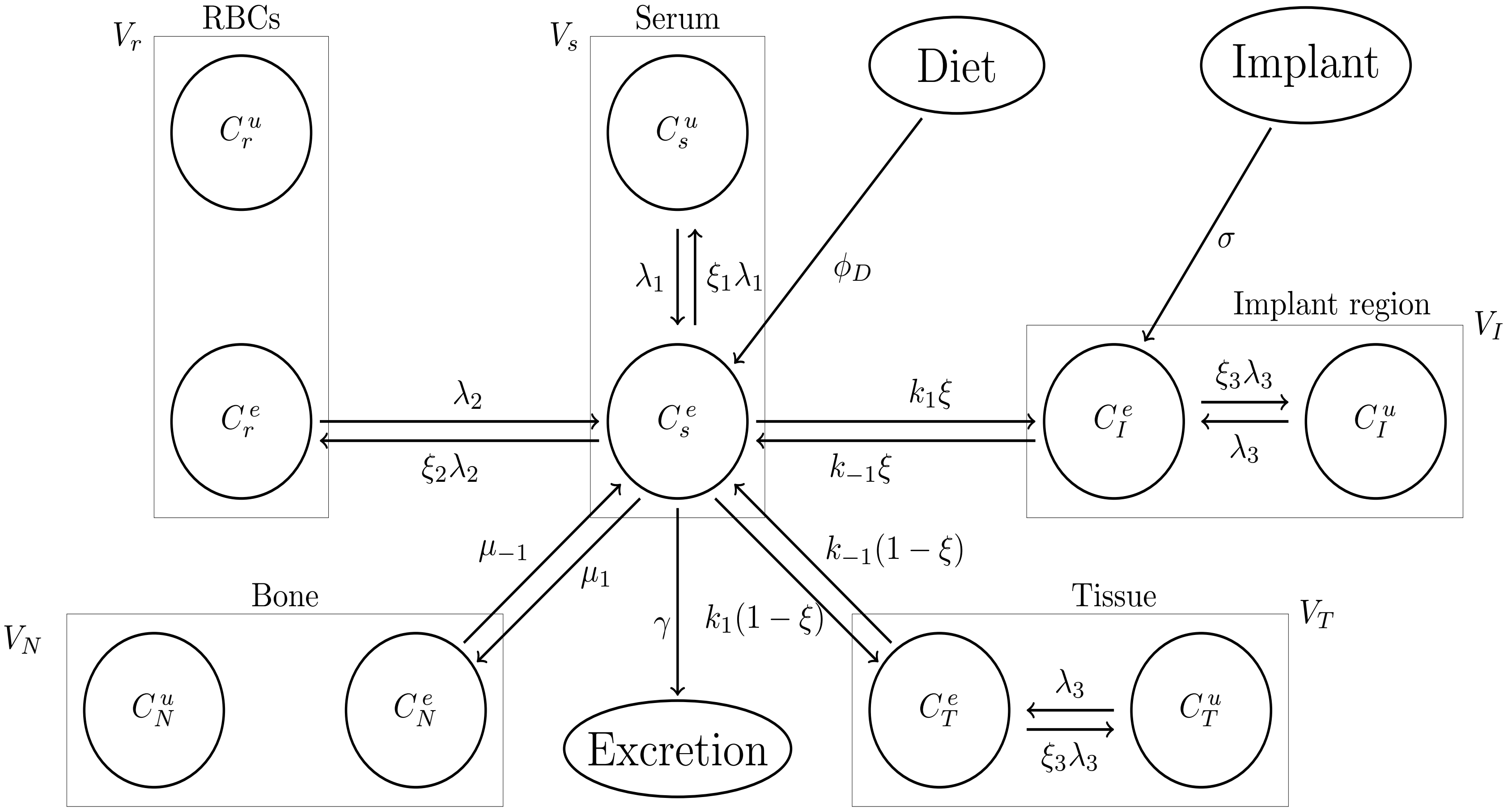

Figure S1: Pathway diagram showing Magnesium exchange to and from the transferable (superscript e) and nono-transferable (superscript u) concentrations in the serum $\boldsymbol{C_s}$, RBCs $\boldsymbol{C_r}$, bone $\boldsymbol{C_N}$, tissue $\boldsymbol{C_T}$ and implant $\boldsymbol{C_I}$ in a well-mixed tissue compartment system where $\xi = V_I/V_{T_{tot}}$. The full model discussed in Supplementary information A is derived from this diagram and then simplified using ideas described in Supp. Info. A.1 leading to equations (1)-(4).

exposed and unexposed bone. We note that the bone is acting as a passive absorber of Mg(II) ions above homoeostatic levels, with no active adaptive process being described. For tissues, it is assumed that there is an exchange between exposed and unexposed forms, with partitioning ξ_3 and exchange rate constant λ_3. Only the exposed form in tissues and serum can be exchanged between the compartments (with rate constants k_1 and k_{-1}).

The PBPK equations are derived using mass balance principles and the simplest kinetics for each of the pathways illustrated in Fig. S1. For the blood compartment we have

$$\begin{aligned}
V_s \frac{dC_s^e}{dt} &= \phi_D - \gamma C_s^e - \mu_1 C_s^e + \mu_{-1} C_N^e - k_1 C_s^e + k_{-1}((1-\xi) C_T^e + \xi C_I^e) \\
&\qquad + \lambda_1 (C_s^u - \xi_1 C_s^e) + \lambda_2 (C_r^e - \xi_2 C_s^e), \\
V_s \frac{dC_s^u}{dt} &= -\lambda_1 (C_s^u - \xi_1 C_s^e), \\
V_r \frac{dC_r^e}{dt} &= -\lambda_2 (C_r^e - \xi_2 C_s^e), \\
V_r \frac{dC_r^u}{dt} &= 0,
\end{aligned} \tag{A.1}$$

for the bone compartment

$$\begin{aligned}
\phi V_N \frac{dC_N^e}{dt} &= \mu_1 C_s^e - \mu_{-1} C_N^e, \\
(1-\phi) V_N \frac{dC_N^u}{dt} &= 0,
\end{aligned} \tag{A.2}$$

for the tissue compartment

$$\begin{aligned}
V_T \frac{dC_T^e}{dt} &= (1-\xi)(k_1 C_s^e - k_{-1} C_T^e) + \lambda_3 (C_T^u - \xi_3 C_T^e), \\
V_T \frac{dC_T^u}{dt} &= -\lambda_3 (C_T^u - \xi_3 C_T^e),
\end{aligned} \tag{A.3}$$

and for the implant zone

$$\begin{aligned}
V_I \frac{dC_I^e}{dt} &= \sigma + \xi (k_1 C_s^e - k_{-1} C_I^e) + \lambda_3 (C_I^u - \xi_3 C_I^e), \\
V_I \frac{dC_I^u}{dt} &= -\lambda_3 (C_I^u - \xi_3 C_I^e),
\end{aligned} \tag{A.4}$$

where

$$\xi = \frac{V_I}{V_{T_{tot}}} \tag{A.5}$$

is the fraction of tissue directly affected by the implant, with $V_{T_{tot}} = V_T + V_I$. We note it is difficult to be precise on a value for V_I, however, it turns out that it makes little impact on the key results in the clinically relevant case of $\xi \ll 1$. All the constant parameters are listed in Tables 1 and S1 with descriptions and estimates for their values. A full discussion of these estimates is given in Supplementary information B representing values for a "typical", healthy adult and are used as the "standard set" in this study for proof of principle purposes.

On instalment of the implant, it is expected that Mg levels in each of the compartments are in their homoeostatic state (indicated with overbars) namely

$$C_s^j(0) = \overline{C}_s^j, \quad C_r^j(0) = \overline{C}_r^j \quad C_N^j(0) = \overline{C}_N^j, \quad C_T^j(0) = \overline{C}_T^j, \quad C_I^j(0) = \overline{C}_T^j, \tag{A.6}$$

where $j = \{e, u\}$, noting that $\overline{C}_N^u = \overline{C}_N^e$ is assumed. Equations (A.1) and (A.2) imply that C_r^u and C_N^u are constant, so $C_r^u = \overline{C}_r^u$ and $C_N^u = \overline{C}_N^u$.

The total quantity of Mg in the body, M is given by,

$$M = V_s(C_s^e + C_s^u) + V_r(C_r^e + C_r^u) + V_N(\phi C_N^e + (1-\phi)C_N^u) + V_T(C_T^e + C_T^u) + V_I(C_I^e + C_I^u), \tag{A.7}$$

and equations (A.1)-(A.4) yields on summation,

$$\frac{dM}{dt} = \phi_D + \sigma - \gamma C_s^e. \tag{A.8}$$

In the absence of an implant, $\sigma = 0$, and at homoeostasis we have $\overline{C}_s^e = \phi_D/\gamma$ in which the intake rate ϕ_D is in balance with the excretion rate $\gamma \overline{C}_s^e$; this will be the assumed state when the implant is introduced in the simulations. With an implant, the serum Mg concentration

will eventually settle to $C_s^e = (\phi_D + \sigma)/\gamma$, so that the implant is predicted to increase serum concentration by $100\sigma/\phi_D$ percent; however, as will be shown, this level may not be reached during the life time of the implant. Given that C_r^u and C_N^u are constant, the current system consists of eight linear ordinary differential equations (ODEs).

A.1 Model reduction

The above model describes Mg interchange within compartments (expected to be a relatively rapid process, equilibrating over a timescale of O(secs-mins)), between compartments (slower, O(hours-days)), vascular build-up and excretion (slower, O(hours-days)) and implant degradation (slowest O(months-years)); the disparity in timescales can be exploited to simplify the model. We are particularly interested in how the process of Mg release and Mg regulation in the blood effects the systemic levels of Mg in the long-term. Focussing on events on the intermediate timescale (hours/days), the processes in the faster timescale are assumed to be in a state of quasi-equilibrium, so that Mg concentration within each compartment satisfies

$$C_s^u \sim \xi_1 C_s^e, \qquad C_r \sim \xi_2 C_s^e, \qquad C_T^u \sim \xi_3 C_T^e, \qquad C_I^u \sim \xi_3 C_I^e. \tag{A.9}$$

Adding equations (A.1)-(A.4) and using these relationships, the unexposed compartments decouple leading to the reduced form of the model for the exposed compartments

$$(V_s(1+\xi_1) + V_r\xi_2)\,\frac{dC_s}{dt} = \phi_D - (\gamma + \mu_1 + k_1)\,C_s + \mu_{-1}\,C_N + k_{-1}\,((1-\xi)C_T + \xi C_I), \tag{A.10}$$

$$\phi\, V_N\,\frac{dC_N}{dt} = \mu_1\,C_s - \mu_{-1}\,C_N, \tag{A.11}$$

$$V_T(1+\xi_3)\,\frac{dC_T}{dt} = (1-\xi)\,(k_1 C_s - k_{-1} C_T), \tag{A.12}$$

$$V_I(1+\xi_3)\,\frac{dC_I}{dt} = \sigma + \xi\,(k_1 C_s - k_{-1} C_I), \tag{A.13}$$

where the subscript "e" has been dropped for simplicity. This reduction process is described more formally in C.1 using non-dimensionalisation (this is a necessary step for the timescale analysis discussed in Section C.3 and that summarised in Section 2.1.2). For completeness, the initial conditions used in the simulations to follow reflect pre-treatment homoeostasis, i.e.

$$C_s = \overline{C}^e_s, \quad C_N = \overline{C}^e_N, \quad C_T = \overline{C}^e_T, \quad C_I = \overline{C}^e_T, \tag{A.14}$$

at $t = 0$, where $\overline{C}^e_s = \phi_D/\gamma, \overline{C}^e_N = \mu_1 \overline{C}^e_s/\mu_{-1}$ and $\overline{C}^e_T = k_1 \overline{C}^e_s/k_{-1}$. The reduced system consists of four linear ODEs describing events on a timescale of O(hours) and beyond.

For Section 2.2.3, the model is modified to consider very simply dietary control to counteract against rising systemic Mg levels via dietary control in response to implant biodegredation. This is done by modifying dietary intake rate (A.10) from ϕ_D to $\rho\phi_D$, giving

$$(V_s(1+\xi_1) + V_r\xi_2)\frac{dC_s}{dt} = \rho\phi_D - (\gamma + \mu_1 + k_1)\, C_s + \mu_{-1}\, C_N + k_{-1}\,((1-\xi)C_T + \xi C_I), \tag{A.15}$$

such that $\rho = 1$ represents "normal" intake and $\rho = 0$ is a zero dietary intake of Mg.

Supplementary information B : Parameterization

The parameters in the model will vary considerably across individuals, depending on age, sex, race, weight, state of health, medication etc. Outlined in this Supporting Information section is the derivation of the values listed in Tables 1 and S1, relevant for an "average", healthy adult human. However, the values are based on studies from a number of different sources, and within the same source there is a high degree of variability. Hence, the parameter values derived are intended to be representative rather than definitive, in the hope that the "specific values" for most healthy individuals lie within a few percent of those quoted in Tables 1 and S1; the **finally selected values are indicated in boldface**.

Studies on magnesium physiology and hypermagnesemia were investigated to determine approximate values for the parameters in the model. Healthy levels of plasma Mg are recognised to be in the range 0.65 - 1.05 mmol/L, more than this is refered to as hypermagnesemia. Concentrations above 3 mmol/L can be problematic, and above 7 mmol/L can lead to cardiac arrest [1].

Data for the amount of Mg in the human body is listed in Tables S2, taken from [1,2,7], and the amount which is exchangeable is shown in Table S3 [1,2]. The data in Table S2 are typical levels found in an average 70 kg adult human body, totalling 1000.7 mmol of magnesium. In the model, muscle, soft tissue and liver are bundled together as "tissue"; the data in Table S2 indicates that the concentrations of Mg in the major organs are not too dissimilar. For tissues and blood the exposed and unexposed Mg is assumed to be well mixed within their volumes, whilst in bone we assume there are distinct regions of exposed (volume fraction $\boldsymbol{\phi} = e_N = \mathbf{0.3}$) and unexposed, each having the same Mg concentration at homoeostasis. **The homoeostatic levels of exposed, $\overline{\boldsymbol{C}}_{\boldsymbol{i}}^{\boldsymbol{e}}$, and unexposed, $\overline{\boldsymbol{C}}_{\boldsymbol{i}}^{\boldsymbol{u}}$, Mg** is calculated from the data in Table S3 using

$$\overline{C}_i^e = \frac{e_i\, M_i}{V_i}, \quad \overline{C}_i^u = \frac{(1-e_i)\, M_i}{V_i}, \quad \overline{C}_N^e = \overline{C}_N^u = \frac{M_N}{V_N}, \tag{B.1}$$

Table S2: Distribution of magnesium in the average adult human being[7]. †Recorded as 2.6[7], adjusted to three significant figures. *Recorded as 43.2[7], corrected here so the values for the bone are consistent.

Tissue	Weight (kg wet wt)	Concentration (mmol/kg wet wt)	Content (mmol)	% of total body magnesium
Serum	3.0	0.85	2.55†	0.3
Red blood cells	2.0	2.5	5.0	0.5
Soft tissue	22.7	8.5	193.0	19.3
Muscle	30.0	9.0	270.0	27.0
Bone	12.3	43.1*	530.1	52.9
TOTAL	70.0		1000.7	100

Table S3: Filtered version of data in Table S2 (columns 2 and 3) and fraction of exchangeable Mg in serum, RBCs, bone and tissue (column 4) sourced from †[2] and ‡[9].

Location	Volume (V_i, L)	Content (M_i, mmol)	Exposed Mg fraction (e_i)
Serum	3.0	2.55	0.65†
RBCs	2.0	5	0.1‡
Tissue	52.7	463	0.25†
Bone	12.3	530.1	0.3†

where $i = \{s, r, T\}$. The resulting values are recorded in Tables 1 and S1. **There appears to be no data for the exchange rates $\boldsymbol{\lambda_1, \lambda_2}$ and $\boldsymbol{\lambda_3}$**, but it is assumed that these are significantly faster than the equivalent parameters across compartments. Using this assumption, the equilibration between exposed and unexposed concentrations happens much faster than the other processes in the system, from which we can deduce the partition coefficients as $\boldsymbol{\xi_1} = \overline{C}_s^u/\overline{C}_s^e = (1 - e_s)/e_s \approx \mathbf{0.538}$, $\boldsymbol{\xi_2} = \overline{C}_r^e/\overline{C}_s^e = e_r M_r V_s / e_s M_s V_r \approx \mathbf{0.452}$ and $\boldsymbol{\xi_3} = (1 - e_T)/e_T = \mathbf{3}$ (see Section C.1.1 for a more rigorous justification of these estimates and Section 2.1.1 for the reduced model resulting from this). Using the data in Tables 1 and S1, the **measured homoeostatic serum concentration of Mg is $\boldsymbol{C_{hom} = \overline{C}_s^e + \overline{C}_s^u \approx 0.85}$ mmol/L**, roughly in the middle of the healthy 0.65-1.05 mmol/L range; **hypermagnesemia is thus characterised by a $\boldsymbol{C_{hyp}/C_{hom} = 1.05/0.85 \approx 1.24}$** fold increase in serum Mg concentration and that of the **problematic case is $\boldsymbol{C_{sev}/C_{hom} = 2.9/0.85 \approx 3.41}$**[8].

The average dietary intake of Mg by healthy humans is about 15 mmol (or 360 mg) per

day[1,10]. There is an exchange of Mg between the intestine and blood supply, where a net exchange of 4 mmol (or 100 mg, i.e. about 28% is absorbed) into the blood is reported[8,10]; this suggests $\phi_D \approx 4$ mmol/day. However, isotope tracer studies suggest that the absorption rate is between 35-55% (mean around 44%)[11,12] of ingested Mg, suggesting that $\phi_D = 6.5$ mmol/day. An alternative means to estimate Mg intake ϕ_D is to measure its excretion rate. The kidneys are mostly responsible for the excretion of excess magnesium in the urine and, when healthy, can increase and decrease the excretion rate depending on plasma levels[1]; this process is supplemented by excretion into the intestines at a considerably lesser extent. To maintain homeostasis, the excretion rate will be roughly that of Mg intake via the intestines (i.e. $\gamma \overline{C}_s^e \approx \phi_D$), and is reported to be around 4 mmol/day[1,2,8,10] and slightly higher in the isotope tracer studies[11–13]. In selecting the parameters ϕ_D, γ, together with the exchange rate parameters μ_* and k_* (discussed further below), simulations were undertaken using a modified version of system (1)-(4) presented in Section A.1; in particular, to find parameters that yield solutions which are recognisably close to those in the tracer studies of[11,12]. Denoting the Mg isotope tracer concentrations with a †, the dimensional equations for the exposed concentration of Mg are

$$(V_s(1+\xi_1) + V_r\xi_2)\,\frac{dC_s^\dagger}{dt} = -\,\gamma\, C_s^\dagger - \mu_1\, C_s^\dagger + \mu_{-1}\, C_N^\dagger - k_1\, C_s^\dagger + k_{-1}\, C_T^\dagger, \tag{B.2}$$

$$\phi\, V_N\,\frac{dC_N^\dagger}{dt} = \mu_1\, C_s^\dagger - \mu_{-1}\, C_N^\dagger, \tag{B.3}$$

$$V_T(1+\xi_3)\,\frac{dC_T^\dagger}{dt} = k_1 C_s^\dagger - k_{-1} C_T^\dagger, \tag{B.4}$$

$$\frac{dM_u^\dagger}{dt} = -\,\gamma\, C_s^\dagger \tag{B.5}$$

subject to $(V_s(1+\xi_1) + V_r\xi_2)C_s^\dagger(0) = M_0$ and $C_N^\dagger(0) = C_T^\dagger(0) = M_u^\dagger(0) = 0$, where $M_u^\dagger$ is the total excreted Mg (urine and faeces) in mmol and M_0 is the number of mmol of intravenously introduced Mg; the system being linear means that there is no interaction between tracer and non-tracer Mg. Fig. S2 shows the evolution of Mg in the compartments in terms of percentage of the initial quantity of Mg tracer. Here, the parameters are chosen so that

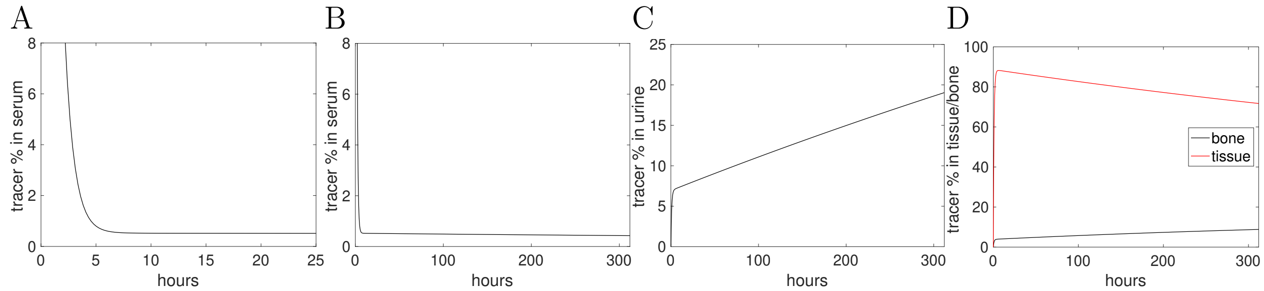

Figure S2: Plots show the simulated tracer percentage using equations (B.2)-(B.5) and the parameters of Table 1; plots A and B show the percentage in serum, C the percentage in urine and D in tissue and bone. The results agree reasonably well to those shown in Fig. 2(A)-(C) of[11] for the normal diet case (i.e. the 800 mg calcium/day case).

the solutions matched reasonably well to the results of Sojka *et al.*[11], in particular the bend in the curve in the Fig.S2A (to occur at about 5 hours, driven by μ_* and k_*, see below), a rapid initial jump in percentage urine to about 6-8% and the slope of the settled urine profile (Fig.S2C); we note several investigations were undertaken Sojka *et al.*[11] (different diets and Mg ion delivery), we chose the $^{25}Mg^{2+}$ tracer data, where a single 20mg dose was administered intravenously. To capture the initial jump, we set $\boldsymbol{\phi_D = 6\,\mathrm{mmol/day}}$ (hence $\boldsymbol{\gamma \approx 10.9\,\mathrm{L/day}}$). The serum and urine profiles in Figs.S2B and C matches well to that reported[11,12] (particularly qualitatively), though the excretion rate from days 1-13 is underestimated by the model, i.e. $1\%\,\mathrm{day}^{-1}$ is predicted as opposed to about $1.5\%\,\mathrm{day}^{-1}$ reported[11]. Fig.S2D shows the extent of Mg that infiltrates tissue and bone, showing that a large amount of ingested Mg is initially taken up by tissue; however, as the initial intravenous bolus consists of around 20 mg of Mg, the rise of Mg in these compartments is $< 1\%$ overall.

The specific mass exchange rates of magnesium between blood and tissue, k_1, and between blood and bone, μ_1, appear not to be reported in the literature. A few studies derive kinetic values for unspecified compartments[11–13], which are therefore not directly applicable here. Brown *et al.*[14] provides a plethora of reference values for PBPK parameters used in pharmacological models; they are not compound specific, but these values have been used in metal ion PBPK studies, e.g. for zinc oxide[15] and for manganese[16]. The blood flow rates are identified as fractions of cardiac output, which is well known to be 5 L/min for

an average 70 kg human being. Here 4.2% of the cardiac output is reported to flow to the bone, while the rest flows to the tissues[14]; this yields $k_{1_{brown}} \approx 6897.6\,\text{L/day}$ and $\mu_{1_{brown}} \approx 302.4\,\text{L/day}$. For the k_{-1} and μ_{-1} values, the homoeostatic state predicted by the model in Section 2.1.1 is used, from which it can be deduced $\boldsymbol{k_{-1}/k_1 \sim \overline{C}_s^e/\overline{C}_T^e \approx 0.252}$ and $\boldsymbol{\mu_{-1}/\mu_1 \sim \overline{C}_s^e/\overline{C}_N^e \approx 0.0385}$ (recalling overbars represent homoeostatic concentrations). However, using these values for k_1 and μ_1 yield solutions of (B.2)-(B.5) that poorly predict the isotope tracer data. Experimenting with the parameters using $k_1 = \alpha k_{1_{brown}}$ and $\mu_1 = \alpha \mu_{1_{brown}}$, we obtained the results in Fig. S2, that agree reasonably with Sojka *et al.*[11], using $\alpha = 0.02$. Hence, we choose $\boldsymbol{k_1 \approx 138\,\text{L/day}}$ and $\boldsymbol{\mu_1 \approx 6.05\,\text{L/day}}$, with k_{-1} and μ_{-1} fixed by the above ratios.

The degradation of Mg implants (typically screws, pins and stents) and its alloys has been investigated both *in vitro* and *in vivo* by a number of investigators. The papers typically do not provide degradation rate values, so we have to make rough estimates based on the papers' content; however, a good estimate of the order of magnitude is sufficient for this paper. In Wang *et al.*[17], they tracked the corrosion of a Mg pin in a rats femur that originally was 1.2 mm in diameter and 25 mm in length (ca $0.028\,\text{cm}^3$). Following an initial phase of 2 weeks in which 10% of the original volume was lost (see Fig. 1F in supplementary material of Wang *et al.*[17]), a further 5% was lost over the next 6 weeks; this represents about $\sigma_{rat} \approx 0.015\,\text{mmol/day}$ for the first 2 weeks and $\sigma_{rat} \approx 0.003\,\text{mmol/day}$ for the next 6 weeks. Scaling up to human proportions, a typical screw for clinical use has dimensions of 3.2mm in diameter and 32mm in length, will have about 4× the surface area, so we estimate the σ_{rat} values to translate to $\sigma \approx 0.06\,\text{mmol/day}$ and $\sigma \approx 0.01\,\text{mmol/day}$ for the 2 week and 6 week phases in humans, respectively. The data shown in Wang *et al.*[17] lacks values between weeks 2 and 8 and it is unclear what the true trajectory of the curve will be. For their *in vitro* study, they inserted 1.2mm diameter and 10 mm long pin in a cell culture medium and used pH and H_2 volume as a proxy for the corrosion. The results (see Fig. 1H) suggests that after 120 hours around $0.45\,\text{cm}^3$ of H_2 is produced. Applying

the ideal gas law, assuming a temperature of 310 K (the paper did not say), this equates to 0.017 mmol of H_2 gas produced and hence corroded Mg, suggesting a corrosion rate of around 0.003 mmol/day (as a 1.2×10 mm pin has at most one tenth of the surface area of a 3.2×32 mm screw, this translates in human dimension to $\sigma \approx 0.003 \times 10 \approx 0.03$ mmol/day). A similar *in vitro* experiment was undertaken by Sato *et al.*[18] using $10 \times 10 \times 1$ mm slabs of two Mg-Zn alloys immersed in 15 ml of medium, Mg(II) ion concentration increased by about 0.08 mg/cm^3 (1.57% Zn alloy) and 0.12 mg/cm^3 (14.2% Zn alloy), indicating corrosion rates of around $\sigma \approx 0.05 - 0.07$ mmol/day. They also described an *in vivo* investigation involving a $30 \times 10 \times 1$ mm plate in the backs of rats; although no explicit data is given on degradation rate for this case, using the given measurements for urine content, suggest $\sigma_{plate} \approx 0.05 - 0.07$ mmol/day (though the implant is huge relative to the size of the rat, the surface area is around that of a screw for a human implant). For the simulation shown in Fig. 4, as humans are O(200) times the mass of rats, the surface area of a proportionately scaled up plate of 1 mm has O(200) times the surface area, $\sigma \approx 200 \times 0.05 = 10$ mmol/day (0.05 mmol/day being used as the standard release rate, see below); note the a plate of the same aspect ratio would be $\sqrt{200} \times (30 \times 10) \times 1 \approx 424 \times 141 \times 1$ mm ($\equiv 0.06$ L). Zheng *et al.*[19] studied Mg implant corrosion in rats with partial nephrectomy (5/6 of kidney mass removed), used as a chronic kidney disorder model. They used a 1.2 mm diameter and 25 mm length pure Mg and Mg-Sr-Zn pins. The pure Mg pin mass dropped by about 20% drop over 12 weeks, giving a corrosion rate of $\sigma \approx 0.05$ mmol/day, whilst the alloy pin was about 90% degraded by 9 weeks, representing a corrosion rate of $\sigma \approx 0.3$ mmol/day; the latter seems to be an outlier, where perhaps the author chosen this alloy as a means to impart a relatively rapid release of Mg into the animal. In Biber *et al.*[20], a 3.2×32 mm implanted in the humerus had more or less completely vanished within 12 months, suggesting a degradation rate of $\sigma \gtrsim 0.05$ mmol/day. In another study, Lee *et al.*[4] recorded that a 2.3×14 mm screw had degraded completely between 6-12 months, giving $0.011 < \sigma < 0.023$ mmol/day. In their paper they also showed, using micro CT that an 2×8 mm Mg in a rabbit showed

a fairly uniform degradation in the first 6 months at a rate of $\sigma_{rabbit} \approx 0.004\,\text{mmol/day}$ (scaling up to a $3.2 \times 32\,\text{mm}$ screw, this relates to about $\sigma \approx 0.04\,\text{mmol/day}$). This work suggests that assuming a constant σ is not unreasonable. It is clear that the Mg degradation rate σ is dependent on the alloy, setting and size of implant. Nevertheless, for a human relevant implant, a σ lying between 0.01-0.1 mmol/day seems reasonable, hence the choice of $\boldsymbol{\sigma} \approx \mathbf{0.05\,mmol/day}$ in the "standard parameter set".

Since the tissue of the implant zone is not clearly defined, the value of V_I is arbitrary. The value of V_I is chosen so that $V_I/V_T = 0.0001$ giving $\boldsymbol{V_I} \approx \mathbf{5.27\,ml}$, roughly 20 times the volume of a $3.2 \times 32\,\text{mm}$ screw. Amongst the *in vivo* measurements made Sato *et al.*[18] was the concentration of Mg(II) ions in the sub-cutaneous tissue in the vicinity of a $30 \times 10 \times 1\,\text{mm}$ implant, which is scaled up to an equivalent slab of $424 \times 141 \times 1\,\text{mm}$ (volume $V_{slab} \approx 0.06\,\text{L}$) in human proportions for the simulations shown in Fig. 4 in this paper. Here, choosing $V_I = 20V_{slab}$, the simulations predict an $8\times$ increase in Mg concentration local to the implant site, marginally higher than, but comparable to, the 2-6$\times$ increase measured in subcutaneous tissue. Larger V_I will lower the predicted C_I, but perhaps less valid in biologically relevant geometries. An alternative calculation based on Zhang *et al.*[21] suggests a diffusion distance of 3-4 mm, from which a "diffusion-region"cylinder with extending 3 mm all around a $3.2 \times 32\,\text{mm}$ screw has a volume about 10 times that of the screw itself, which is consistent with above estimates. Regardless of the most appropriate value of V_I, in physiologically relevant cases of $V_I \ll V_T$, the value of V_I has very little effect on the overall results.

Supplementary information C : Mathematical analysis

C.1 Non-dimensionalisation of the full pathway model

The model reduction outlined in Section A.1 of Supplementary information A can be done using timescale separation arguments. In this section a more formal discussion is presented following non-dimensionalising the model. The main benefit of this is to facilitate the timescale analysis presented in C.3. The model equations (A.1)-(A.4) describe events over a number of timescales, e.g. timescale for equilibration within a compartment (assumed fast at O(secs/mins)), between compartments (slower at O(mins/hours)), timescale for excretion (slower at O(hours/days)) and implant occupation (slowest at O(months/years)). In what follows we scale time using

$$t = \frac{V_s}{k_1}\widehat{t},$$

where the factor V_s/k_1 is the Mg absorption timescale from blood into tissue, which using the data in Table 1 implies that $\widehat{t} = 1$ represents just over 30 minutes. This scaling means that events on an intermediate timescale occur at $\widehat{t} = O(1)$, fast events when $t \ll 1$ and slower events when $t \gg 1$. The concentrations are rescaled as follows

$$\begin{aligned}
C_s^e &= \overline{C}_s^e\, C_s^*, \quad C_r^e = \overline{C}_s^e\, C_r, \quad C_N^e = \frac{\mu_1}{\mu_{-1}}\overline{C}_s^e\, C_N^*, \quad C_T^e = \frac{k_1}{k_{-1}}\overline{C}_s^e\, C_T^*, \quad C_I^e = \frac{k_1}{k_{-1}}\overline{C}_s^e\, C_I^*,\\
C_s^u &= \overline{C}_s^e\, \widehat{C}_s^u, \quad C_r^u = \overline{C}_s^e\, \widehat{C}_r^u, \quad C_N^u = \frac{\mu_1}{\mu_{-1}}\overline{C}_s^e\, \widehat{C}_N^u, \quad C_T^u = \frac{k_1}{k_{-1}}\overline{C}_s^e\, \widehat{C}_T^u, \quad C_I^u = \frac{k_1}{k_{-1}}\overline{C}_s^e\, \widehat{C}_I^u,
\end{aligned}$$

noting that the dimensionless exposed Mg concentrations C_j^* are identical to the scaled concentrations discussed in Sections 2.1.2 and 2.2. The dimensionless form of the parameters

are

$$S = \frac{\sigma V_s k_{-1}}{\overline{C}_s^e V_I k_1^2}, \quad \mu = \frac{\mu_1}{k_1}, \quad \Gamma = \frac{\gamma}{k_1} = \frac{\phi_D}{\overline{C}_s^e\, k_1}, \quad \xi = \frac{V_I}{V_{T_{tot}}}, \quad \xi_B = \frac{V_r}{V_s}, \quad \xi_N = \frac{V_s \mu_{-1}}{V_N \mu_1},$$
$$\xi_T = \frac{V_s k_{-1}}{V_T k_1}, \quad \frac{1}{\epsilon} = \frac{\lambda_1}{k_1}, \quad \frac{\widehat{\lambda}_2}{\epsilon} = \frac{\lambda_2}{k_1}, \quad \frac{\widehat{\lambda}_{3_1}}{\epsilon} = \frac{V_s \lambda_3}{V_T k_1}, \quad \frac{\widehat{\lambda}_{3_2}}{\epsilon} = \frac{V_s \lambda_3}{V_I k_1}. \tag{C.1}$$

Dropping the hats for clarity, the following system is attained, for the blood compartment

$$\begin{aligned}
\frac{dC_s^*}{dt} &= \Gamma\,(1 - C_s^*) \,+\, \mu\,(C_N^* - C_s^*) \,+\, (1-\xi)\,(C_T^* - C_s^*) \,+\, \xi\,(C_I^* - C_s^*) \\
&\quad + \frac{1}{\epsilon}(C_s^u - \xi_1 C_s^*) \,+\, \frac{\lambda_2}{\epsilon}\,(C_r - \xi_2 C_s^*), \\
\frac{dC_s^u}{dt} &= -\,\frac{1}{\epsilon}\,(C_s^u - \xi_1 C_s^*), \\
\xi_B \frac{dC_r}{dt} &= -\,\frac{\lambda_2}{\epsilon}(C_r - \xi_2 C_s^*),
\end{aligned} \tag{C.2}$$

for the bone compartment

$$\phi\,\frac{dC_N^*}{dt} = \mu\,\xi_N\,(C_s^* - C_N^*), \tag{C.3}$$

for tissue compartment

$$\begin{aligned}
\frac{dC_T^*}{dt} &= (1-\xi)\,\xi_T\,(C_s^* - C_T^*) \,+\, \frac{\lambda_{3_1}}{\epsilon}\,(C_T^u - \xi_3 C_T^*), \\
\frac{dC_T^u}{dt} &= -\,\frac{\lambda_{3_1}}{\epsilon}(C_T^u - \xi_3 C_T^*),
\end{aligned} \tag{C.4}$$

and for the implant zone

$$\begin{aligned}
\frac{dC_I^*}{dt} &= S \,+\, (1-\xi)\,\xi_T\,(C_s^* - C_I^*) \,+\, \frac{\lambda_{3_2}}{\epsilon}\,(C_I^u - \xi_3 C_I^*), \\
\frac{dC_I^u}{dt} &= -\,\frac{\lambda_{3_2}}{\epsilon}(C_I^u - \xi_3 C_I^*).
\end{aligned} \tag{C.5}$$

Table S4: Dimensionless parameters in the model for healthy individuals, which are derived using data in Table 1. †Values change depending on kidney function (Γ) and size and/or quantity of implants (ξ).

Parameter	Value	Parameter	Value	Parameter	Value
S	0.0939	μ	0.0438	ϕ	0.3
ξ_B	0.667	ξ_T	0.0143	ξ_N	0.00313
ξ_1	0.538	ξ_2	0.452	ξ_3	3.00
$\Gamma^\dagger$	0.0787	$\xi^\dagger$	0.0001		

The dimensionless initial conditions representing the homoeostatic state are

$$\begin{gathered} C_s^*(0) = C_N^*(0) = C_T^*(0) = C_I^*(0) = 1, \quad C_r(0) = \frac{\overline{C}_r^e}{\overline{C}_s^e}, \\ C_s^u(0) = \frac{\overline{C}_s^u}{\overline{C}_s^e}, \quad C_T^u(0) = C_I^u(0) = \frac{k_{-1}\overline{C}_T^u}{k_1\overline{C}_s^e}, \end{gathered} \tag{C.6}$$

and dimensionless variables $C_r^u = \overline{C}_r^u/\overline{C}_s^e$ and $C_N^u = \mu_1\overline{C}_N^u/\mu_{-1}\overline{C}_s^e$ are constant.

C.1.1 Formal reduction of the full model

It is assumed that the timescale for Mg exchange within each compartment is much faster than that across compartments, which means that $\epsilon \ll 1$ and that fast and slow processes can be "lumped" together as described in the introduction. The dynamics of the slower processes, which is of interest here, can be derived directly from (C.2)-(C.5) in the limit $\epsilon \to 0$, whereby at leading order,

$$C_s^u \sim \xi_1 C_s^*, \qquad C_r \sim \xi_2 C_s^*, \qquad C_T^u \sim \xi_3 C_T^*, \qquad C_I^u \sim \xi_3 C_I^*, \tag{C.7}$$

hence

$$C_s^u + C_s^* + \xi_B C_r \sim (1 + \xi_1 + \xi_B\xi_2)C_s^*, \quad C_T^u + C_T^* \sim (1 + \xi_3)C_T^*, \quad C_I^u + C_I^* \sim (1 + \xi_3)C_I^*$$

as $\epsilon \to 0$. Adding the equations in (C.2), in (C.4) and in (C.5) we derive the reduced system in full

$$\frac{dC_s^*}{dt} = \frac{1}{1+\xi_1+\xi_B\xi_2}\left(\Gamma\,(1-C_s^*) \,+\, \mu\,(C_N^*-C_s^*) \,+\, (1-\xi)\,(C_T^*-C_s^*) \,+\, \xi\,(C_I^*-C_s^*)\right), \tag{C.8}$$

$$\frac{dC_N^*}{dt} = \frac{\mu\,\xi_N}{\phi}\,(C_s^*-C_N^*)\,, \tag{C.9}$$

$$\frac{dC_T^*}{dt} = \frac{(1-\xi)\,\xi_T}{1+\xi_3}\,(C_s^*-C_T^*)\,, \tag{C.10}$$

$$\frac{dC_I^*}{dt} = \frac{1}{1+\xi_3}\,\left(S \,+\, (1-\xi)\,\xi_T\,(C_s^*-C_I^*)\right), \tag{C.11}$$

subject to the initial conditions

$$C_s^*(0) \,=\, C_N^*(0) \,=\, C_T^*(0) \,=\, C_I^*(0) \,=\, 1. \tag{C.12}$$

This system is equivalent to that presented in Section 2.1.1 and Supplementary material section A.1 on reversion to dimensional quantities. The dimensionless parameter values for the reduced model are listed in Table S4 derived from the values in Table 1 using the formulae in (C.1).

It is worth noting that the data in Table S4 indicates that $\Gamma \approx 0.0787$, meaning that the expected timescale of recovery on removal of an implant is $O(1/\Gamma)$, representing about 6-7 hours for a human with healthy kidneys. The effect of reduced kidney function in this model, i.e. leading to a smaller Γ, has the effect of extending the recovery timescale and consequently slower to adapt to any changes in Mg levels. The nondimensional form of the dietary control equation (11) is

$$\frac{dC_s^*}{dt} = \frac{1}{1+\xi_1+\xi_B\xi_2}\left(\Gamma\,(\rho-C_s^*) \,+\, \mu\,(C_N^*-C_s^*) \,+\, (1-\xi)\,(C_T^*-C_s^*) \,+\, \xi\,(C_I^*-C_s^*)\right). \tag{C.13}$$

C.2 Steady-state analysis

In this section we derive and discuss the steady-state results of Section 2.1.2. In the case of a constant Mg intake (from diet) and release rate from the implant, the solutions to (C.8)-(C.11) will eventually reach a steady or equilibrium state. Whilst there are daily fluctuations due to diet, the degradation rate of the Mg implant will be relatively stable over much of its life. Since the daily amount of Mg is small compared to the that in the body, the equilibrium solution will be a good reflection of the long term Mg levels in response to the implant. The analysis below derives the equilibrium state and in demonstrating its uniqueness and stability we will show that the reduced model produces physiologically meaningful results for all time.

Setting the right-hand sides of (C.8)-(C.11) to zero, results with a linear system that determines the equilibrium state $\mathbf{C}_\infty$, given by

$$\mathbf{C}_\infty = (C^*_{s_\infty}, C^*_{N_\infty}, C^*_{T_\infty}, C^*_{I_\infty}) = \Big(1 + \frac{S\,\xi}{\Gamma\,(1-\xi)\,\xi_T},\ C^*_{s_\infty},\ C^*_{s_\infty},\ 1 + \frac{S\,(\Gamma+\xi)}{\Gamma\,(1-\xi)\,\xi_T}\Big); \quad \text{(C.14)}$$

this will be shown below to be the only equilibrium state and that it is asymptotically stable. We note, in the absence of the implant, when $\xi = 0$, we have $(C^*_{s_\infty}, C^*_{N_\infty}, C^*_{T_\infty}) = (1, 1, 1)$ as expected. The steady-states expressed in dimensional terms are given by (8). The formulae shows the extent of Mg increase as a function of the release rate S and kidney function Γ.

The system (C.8)-(C.11), being linear, can be written in the form $U d\mathbf{C}/dt = A\,\mathbf{C} + \mathbf{b}$, where $\mathbf{C}^T = (C^*_s\ C^*_N\ C^*_T\ C^*_I)$, U is a diagonal 4×4 matrix with positive elements equal to $\text{diag}(1+\xi_1+\xi_B\xi_2, \phi, 1+\xi_3, 1+\xi_3)$ and

$$A = \begin{pmatrix} -\Gamma-\mu-1 & \mu & 1-\xi & \xi \\ \xi_N\mu & -\xi_N\mu & 0 & 0 \\ (1-\xi)\xi_T & 0 & -(1-\xi)\xi_T & 0 \\ (1-\xi)\xi_T & 0 & 0 & -(1-\xi)\xi_T \end{pmatrix}, \qquad \mathbf{b} = \begin{pmatrix} \Gamma \\ 0 \\ 0 \\ S \end{pmatrix}; \quad \text{(C.15)}$$

noting that the steady-state $\mathbf{C}_\infty$ satisfies $A\,\mathbf{C}_\infty + \mathbf{b} = \mathbf{0}$. For $\mathbf{C}_\infty$ to be asymptotically stable, i.e. $\mathbf{C} \to \mathbf{C}_\infty$ as $t \to \infty$, it is sufficient for all of the eigenvalues of matrix A to have negative real part (since the elements of U are all positive). The characteristic polynomial of A is a rather unwieldy quartic equation, with a nonzero constant coefficient (equalling $\mu\xi_N\xi_T^2\Gamma(1-\xi)^2$). This implies the determinant of A is non-zero, hence C_∞ is the only solution of the linear system $A\,\mathbf{C}_\infty = -\mathbf{b}$. To deduce stability we apply the Gershgorin circle theorem, which states that for an $n \times n$ matrix A, each eigenvalue λ_i lies within a disc of radius R_i, defined as $R_i = \sum_{j=1;i\neq j}^{n} |A_{ij}|$, centred at A_{ii} in the complex plane; such a disc is called a Gershgorin disc[22]. Applying this to matrix A in (C.15), the circles for each row lies in the negative real half space, though for rows 2-4 the circle touches the origin; hence there is at least one eigenvalue with negative real part with the rest being non-positive. However, with the product of the eigenvalues being positive, the zero eigenvalue possibility can be discarded, hence all eigenvalues must have negative real parts and thus the steady-state is asymptotically stable. Finally, from (C.8)-(C.11), we have $dC_j^*/dt \geq 0$ when $C_j^* = 0$ for $j = \{s, N, T, I\}$, so that $C_j^* \geq 0$ given nonnegative initial conditions. Consequently, the steady-state $\mathbf{C}_\infty$ is globally stable and the concentrations C_j^* are nonnegative for all $t > 0$. These conclusions also apply when the implant is absent ($\xi = 0$).

C.3 Asymptotic (timescale) analysis

In this section we derive the simplified formulae presented and discussed in Sections 2.1.2 and 2.2 (Section C.3.1) and the slightly more complex and accurate formulae (Section C.3.2) that provides the estimate of the drop in systemic Mg concentration in response to reduced dietary intake discussed at the end of Section 2.2.3.

Examination of Figs. 3 and 4 seem to show two phases of Mg(II) ion accumulation, firstly a phase in which the tissue local to the implant site rises relatively rapidly (around 1-10 days) then a longer phase of systemic response (around 10-600 days). To gain insight into these solutions, we will in this section of Supplementary information C analyse the reduced

system of Section C.1.1 in the biologically relevant limit of $\xi \to 0^+$ (i.e. $V_I/V_{T_{tot}} \to 0$) using singular perturbation analysis (or matched asymptotic expansions)[23,24]. We note the system (C.8)-(C.11) is a 4th-order, linear system of ODEs that can be solved directly, but the solutions of the auxiliary equation will, in general, involve roots of intractable combinations of the parameters. The analysis below will yield analytical formulae that are much easier to interpret and apply.

As stated above, we will consider two cases, (1) a formulation that only partly reflects the parameters in Section C.3.1, which produces a remarkably accurate approximation, and (2) a more rigorous and complex formulation summarised in Section C.3.2. Case (1) yields accurate solutions for serum Mg concentration and is noteworthy for the simplicity of solution, but it does fail to capture the lag-time for response in the bone compartment shown in Fig. 4; the formulation of Case (2) captures this. We note other parameter scalings in terms of ξ can be chosen, so the following formulations are not exhaustive.

C.3.1 Simple formulation

This formulation considers the combined limits $\Gamma \to 0^+$ and $\xi \to 0^+$, i.e. in the limit of the intake-excretion equilibration timescale tending to infinity and $V_I \to 0$; for simplicity, rather than considering the dual limits, we write

$$\Gamma = \xi \, \Gamma^*, \tag{C.16}$$

where $\Gamma^* = O(1)$ is assumed in the analysis (although $\Gamma^* \approx 787$ using the data in Table S4). Focussing only on a "small" Γ is rather lacking in mathematical formality as there are other parameters which are smaller and are treated here as $O(1)$ (see Table S4). However, despite this inconsistency, the analysis in this limit does lead to an approximation that captures well the two time-phase structure, yielding simple formulae for C_s^* and C_I^*, in particular, that accurately approximates the solutions of the full model across a large range of parameter

values, provided that $\xi \ll 1$. The good level of accuracy is largely due to the approximations consisting mainly of ratios of parameters that are sufficiently $O(1)$ in the crucial terms (e.g. within the time point expression T_{Mg}), thereby fortuitously mitigating the inaccuracies that would normally result when assuming small parameters to be $O(1)$ using this analytical approach.

Substituting (C.16) into (C.8)-(C.11), yields the system

$$\frac{dC_s^*}{dt} = \frac{1}{1+\xi_1+\xi_B\xi_2}\Big(\xi\,\Gamma^*(1-C_s^*)+\mu(C_N^*-C_s^*)+(1-\xi)(C_T^*-C_s^*)+\xi(C_I^*-C_s^*)\Big), \tag{C.17}$$

$$\frac{dC_N^*}{dt} = \frac{\mu\xi_N}{\phi}(C_s^*-C_N^*), \tag{C.18}$$

$$\frac{dC_T^*}{dt} = \frac{(1-\xi)\,\xi_T}{1+\xi_3}(C_s^*-C_T^*), \tag{C.19}$$

$$\frac{dC_I^*}{dt} = \frac{1}{1+\xi_3}\left(S+(1-\xi)\,\xi_T\,(C_s^*-C_I^*)\right), \tag{C.20}$$

subject to,

$$\text{at } \ t = 0, \qquad C_s^* = C_N^* = C_T^* = C_I^* = 1. \tag{C.21}$$

In the limit $\xi \to 0$ discussed below, there are two timescales of interest, the first of which involves the growth of Mg around the implant site and the second sees the growth of Mg throughout the body.

C.3.1.1 Timescale 1 (first phase), $t = O(1)$

Denoting variables for this timescale with overbars, we write

$$t = \bar{t}, \;\; C_s^* = \overline{C}_s, \;\; C_N^* = \overline{C}_N, \;\; C_T^* = \overline{C}_T, \;\; C_I^* = \overline{C}_I,$$

and expand the dependent variables in powers of ξ as follows

$$\overline{C}_s \sim \overline{C}_s^{[0]} + \xi\,\overline{C}_s^{[1]}, \qquad \overline{C}_N \sim \overline{C}_N^{[0]} + \xi\,\overline{C}_N^{[1]},$$
$$\overline{C}_T \sim \overline{C}_T^{[0]} + \xi\,\overline{C}_T^{[1]}, \qquad \overline{C}_I \sim \overline{C}_I^{[0]} + \xi\,\overline{C}_I^{[1]}, \tag{C.22}$$

as $\xi \to 0^+$. Substituting these expansions into (C.17)-(C.20) yields at leading order ($O(\xi^0)$),

$$\frac{d\overline{C}_s^{[0]}}{d\bar{t}} = \frac{1}{1+\xi_1+\xi_B\xi_2}\left(\mu\left(\overline{C}_N^{[0]} - \overline{C}_s^{[0]}\right) + \overline{C}_T^{[0]} - \overline{C}_s^{[0]}\right), \tag{C.23}$$

$$\frac{d\overline{C}_N^{[0]}}{d\bar{t}} = \frac{\mu\xi_N}{\phi}\left(\overline{C}_s^{[0]} - \overline{C}_N^{[0]}\right), \tag{C.24}$$

$$\frac{d\overline{C}_T^{[0]}}{d\bar{t}} = \frac{\xi_T}{1+\xi_3}\left(\overline{C}_s^{[0]} - \overline{C}_T^{[0]}\right), \tag{C.25}$$

$$\frac{d\overline{C}_I^{[0]}}{d\bar{t}} = \frac{1}{1+\xi_3}\left(S + \xi_T\left(\overline{C}_s^{[0]} - \overline{C}_I^{[0]}\right)\right) \tag{C.26}$$

Applying the initial conditions $\overline{C}_i = 1$ for $i = \{s, N, T, I\}$, we obtain

$$\overline{C}_s^{[0]} = \overline{C}_T^{[0]} = \overline{C}_N^{[0]} = 1, \qquad \overline{C}_I^{[0]} = 1 + \frac{S}{\xi_T}\left(1 - \exp\left(-\frac{\xi_T}{1+\xi_3}\,\bar{t}\right)\right). \tag{C.27}$$

These solutions indicate that there will be a notable rise in implant site ion concentration when $\bar{t} = O((1+\xi_3)/\xi_T)$, settling to a level $C_I \sim 1 + S/\xi_T$ as $\bar{t} \to \infty$; this time point in dimensional form is $T_1 = V_s(1+\xi_3)/k_1\xi_T = V_T(1+\xi_3)/k_{-1}$, as quoted in Table 2. Meanwhile, the ion concentration in the other compartments is relatively unchanged. For "matching" with the solutions of the next timescale, we have $\overline{C}_s^{[0]} = \overline{C}_N^{[0]} = \overline{C}_T^{[0]} = 1$ and $\overline{C}_I^{[0]} = 1 + S/\xi_T$ as $\bar{t} \to \infty$.

Continuing on to the correction terms (superscript [1]), we get the equations in the same form as (C.24)-(C.26) (without the S in the latter), and

$$\frac{d\overline{C}_s^{[1]}}{d\bar{t}} = \frac{1}{1+\xi_1+\xi_B\xi_2}\left(\mu\left(\overline{C}_N^{[1]} - \overline{C}_s^{[1]}\right) + \overline{C}_T^{[1]} - \overline{C}_s^{[1]} + \overline{C}_I^{[0]} - 1\right), \tag{C.28}$$

with $\overline{C}_I^{[0]}$ given by (C.27). Using (C.23)-(C.25), we can eliminate $\overline{C}_N^{[1]}$ and $\overline{C}_T^{[1]}$ to derive

$$a_3 \frac{d^3\overline{C}_s^{[1]}}{d\bar{t}^3} \;+\; a_2 \frac{d^2\overline{C}_s^{[1]}}{d\bar{t}^2} \;+\; a_1 \frac{d\overline{C}_s^{[1]}}{d\bar{t}} \;=\; \mu \xi_N S, \tag{C.29}$$

where

$$a_3 = \phi(1+\xi_3)(1+\xi_1+\xi_B\xi_2), \quad a_1 = \frac{\mu\Gamma^* \xi_N \xi_T}{\chi},$$
$$a_2 = \mu(1+\xi_3)(\phi + \xi_N(1+\xi_1+\xi_B\xi_2)) \;+\; \phi(1+\xi_T(1+\xi_1+\xi_B\xi_2)+\xi_3),$$

and

$$\chi \;=\; \frac{\Gamma^*\, \xi_N\, \xi_T}{\xi_N\,(1+\xi_T(1+\xi_1+\xi_B\xi_2)+\xi_3)+\phi\,\xi_T}, \tag{C.30}$$

subject to $\overline{C}_s^{[1]}(0) = d\overline{C}_s^{[1]}(0)/d\bar{t} = 0$ and $d^2\overline{C}_s^{[1]}(0)/d\bar{t}^2 = S/(1+\xi_3)(1+\xi_1+\xi_B\xi_2)$. The solution to this is somewhat unwieldy, but we note the coefficients of the derivatives in (C.29) are positive so that the auxiliary equation of the complementary function has one zero and two negative (real part) roots. The feature of most interest is that $\overline{C}_s^{[1]} \sim S\chi\,\bar{t}/\xi_T\Gamma^*$, i.e. grows linearly as $\bar{t} \to \infty$. Hence, when $\bar{t} = O(1/\xi)$ these series approximations breakdown as the leading order terms cease to be dominant in the expansions (C.22), indicating a new balance of terms in the timescale $t = O(1/\xi)$.

C.3.1.2 Timescale 2 (second phase), $t = O(\xi^{-1})$

Over this long timescale, the small Mg(II) ion source term $\xi(C_I^* - C_s^*)$ in (C.17) makes an $O(1)$ contribution to the system wide concentration. Denoting variables for this timescale with a "∼" symbol, we write

$$t = \frac{\tilde{t}}{\xi}, \quad C_s^* = \widetilde{C}_s, \quad C_N^* = \widetilde{C}_N, \quad C_T^* = \widetilde{C}_T, \quad C_I^* = \widetilde{C}_I,$$

and expanding the dependent variables in the same fashion as (C.22), yields up to $O(\xi)$

$$\xi\,\frac{d\widetilde{C}_s^{[0]}}{d\tilde{t}} \;=\; \frac{1}{1+\xi_1+\xi_B\xi_2}\left(\mu\left(\widetilde{C}_N^{[0]}-\widetilde{C}_s^{[0]}\right)+\widetilde{C}_T^{[0]}-\widetilde{C}_s^{[0]}\right), \tag{C.31}$$

$$+\;\frac{\xi}{1+\xi_1+\xi_B\xi_2}\left(\Gamma^*\left(1-\widetilde{C}_s^{[0]}\right)+\mu\left(\widetilde{C}_N^{[1]}-\widetilde{C}_s^{[1]}\right)+\widetilde{C}_T^{[1]}-\widetilde{C}_s^{[1]}\;+\;\widetilde{C}_I^{[0]}-\widetilde{C}_T^{[0]}\right),$$

$$\xi\,\frac{d\widetilde{C}_N^{[0]}}{d\tilde{t}} \;=\; \frac{\mu\xi_N}{\phi}\left(\widetilde{C}_s^{[0]}-\widetilde{C}_N^{[0]}\right)\;+\;\xi\,\frac{\mu\xi_N}{\phi}\left(\widetilde{C}_s^{[1]}-\widetilde{C}_N^{[1]}\right), \tag{C.32}$$

$$\xi\,\frac{d\widetilde{C}_T^{[0]}}{d\tilde{t}} \;=\; \frac{\xi_T}{1+\xi_3}\left(\widetilde{C}_s^{[0]}-\widetilde{C}_T^{[0]}\right)\;+\;\xi\,\frac{\xi_T}{1+\xi_3}\left(-\widetilde{C}_s^{[0]}+\widetilde{C}_T^{[0]}+\widetilde{C}_s^{[1]}-\widetilde{C}_T^{[1]}\right), \tag{C.33}$$

$$\xi\,\frac{d\widetilde{C}_I^{[0]}}{d\tilde{t}} \;=\; \frac{1}{1+\xi_3}\left(S+\xi_T\left(\widetilde{C}_s^{[0]}-\widetilde{C}_I^{[0]}\right)\right)\;+\;\xi\,\frac{\xi_T}{1+\xi_3}\left(-\widetilde{C}_s^{[0]}+\widetilde{C}_I^{[0]}+\widetilde{C}_s^{[1]}-\widetilde{C}_I^{[1]}\right), \tag{C.34}$$

whereby matching with the previous timescale's solutions as $\bar{t}\to\infty$ we have

$$\text{at}\;\;\tilde{t}\;=\;0,\qquad \widetilde{C}_s^{[0]}\;=\;\widetilde{C}_N^{[0]}\;=\;\widetilde{C}_T^{[0]}\;=\;1,\;\;\widetilde{C}_I^{[0]}\;=\;1+\frac{S}{\xi_T}. \tag{C.35}$$

At leading order, all of equations (C.31)-(C.34) are in quasi-equilibrium as $\xi\to 0^+$, so that

$$\widetilde{C}_N^{[0]}\;=\;\widetilde{C}_s^{[0]},\qquad \widetilde{C}_T^{[0]}\;=\;\widetilde{C}_s^{[0]},\qquad \widetilde{C}_I^{[0]}\;=\;\widetilde{C}_s^{[0]}\;+\;\frac{S}{\xi_T}, \tag{C.36}$$

consistent with the initial conditions. Substituting (C.36) into (C.31)-(C.34), then making $\widetilde{C}_N^{[1]}$, $\widetilde{C}_T^{[1]}$, $\widetilde{C}_I^{[1]}$ the subject of equations (C.32)-(C.34), respectively, and substituting these into (C.31), yields the differential equation,

$$\frac{d\widetilde{C}_s^{[0]}}{d\tilde{t}} \;=\; \frac{\xi_N\left(S+\Gamma^*\xi_T-\Gamma^*\xi_T\widetilde{C}_s^{[0]}\right)}{\xi_N(1+\xi_T(1+\xi_1+\xi_B\xi_2)+\xi_3)+\phi\xi_T}, \tag{C.37}$$

which, given (C.35), solves to give

$$\widetilde{C}_s^{[0]}\;=\;1\;+\;\frac{S}{\Gamma^*\xi_T}\left(1-e^{-\chi\tilde{t}}\right), \tag{C.38}$$

where χ is defined by (C.30). From (C.36) we have

$$\widetilde{C}_I^{[0]} = 1 + \frac{S}{\xi_T} + \frac{S}{\Gamma^* \xi_T}\left(1 - e^{-\chi \tilde{t}}\right), \tag{C.39}$$

and $\widetilde{C}_N^{[0]} = \widetilde{C}_T^{[0]} = \widetilde{C}_s^{[0]}$.

Over this timescale, the variables eventually reach their steady-states, namely $\widetilde{C}_N^{[0]} = \widetilde{C}_T^{[0]} = \widetilde{C}_s^{[0]} \to 1 + S/\Gamma^*\xi_T$ and $\widetilde{C}_I^{[0]} \to 1 + S/\xi_T + S/\Gamma^*\xi_T$ as $\tilde{t} \to \infty$, and there are no further timescales of interest. These steady-states are the same as those in (C.14) in the limit $\xi \to 0^+$, given (C.16) and $(1-\xi)^{-1} \sim 1$, as to be expected. Although the steady-states are straightforward to determine in this model, the asymptotic analysis reveals that the timescale, $\widetilde{T}_{Mg}$, for notable Mg ion to increase in the blood is $\tilde{t} \approx \widetilde{T}_{Mg} = 1/\chi$. In terms of the original dimensionless time variable, this timescale is

$$t \sim T^*_{Mg} = \frac{1}{\xi\,\chi} = \frac{\xi_N(1 + \xi_T(1 + \xi_1 + \xi_B\xi_2) + \xi_3) + \phi\xi_T}{\Gamma\xi_N\xi_T}, \tag{C.40}$$

as $\xi \to 0^+$, where $T^*_{Mg} = \widetilde{T}_{Mg}/\xi$; the dimensional form in Table 2 results from $T_{Mg} = k_1 T^*_{Mg}/V_s$. We can thus estimate when Mg(II) ions significantly rise in the blood, thereby determine when the early signs of hypermagnesemia may occur.

Using the Mg(II) ion concentration solutions from both these two timescales, we can formulate a so called uniformly asymptotic solutions by constructing a composite solution of each of the variables; this involves adding the leading order approximations and subtracting the limiting solution as $\bar{t} \to \infty$ and $\tilde{t} \to 0$. Doing this, we obtain

$$C_s^* \sim C_N^* \sim C_T^* \sim 1 + \frac{\xi\,S}{\Gamma\,\xi_T}\left(1 - e^{-t/T^*_{Mg}}\right), \tag{C.41}$$

$$C_I^* \sim 1 + \frac{S}{\xi_T}\left(1 - \exp\left(-\frac{\xi_T}{1+\xi_3}\,t\right)\right) + \frac{\xi\,S}{\Gamma\,\xi_T}\left(1 - e^{-t/T^*_{Mg}}\right), \tag{C.42}$$

as $\xi \to 0^+$, where we have reverted to the original Γ using $\Gamma^* = \Gamma/\xi$. These solutions are considerably easier to compute than the original ODE system and as can be seen in Fig. S3

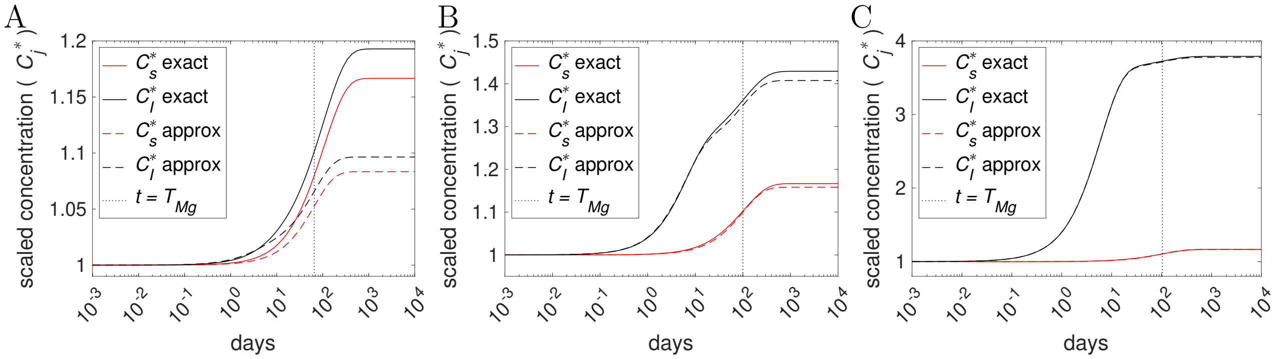

Figure S3: Plots comparing the time dependent solutions of the full model (1)-(4) (solid curves) with the approximations (6) and (7) (dashed curves) for $\xi = 0.5$ (plot A), 0.05 (B) and 0.005 (C) and $\Gamma = (1-\xi)\Gamma^*$ (with $\Gamma^* \approx 787$) for the $n = 20$ case shown in Fig. 3 (chosen to exaggerate the details). The vertical dots indicate $t = T_{Mg}$ given in Table 2. Note the approximations for C_T^* and C_N are visually identical to C_s^*. All other parameters given in Table 1, with V_I, σ, ξ changed according to (10).

are in excellent agreement for small values of ξ and indeed reasonable even at $\xi = 0.5$. The dimensional forms of these equations are given by (6) and (7). The dimensionless form for T_{hyp} is, as $\xi \to 0^+$,

$$T^*_{hyp} \;=\; T^*_{Mg} \ln\left(\frac{S\xi}{S\xi - \Gamma\xi_T(C^*_{hyp}-1)}\right), \tag{C.43}$$

with $S > \Gamma\xi_T(C^*_{hyp}-1)/\xi$; the formula in Table 2 is the dimensional version.

C.3.1.3 Magnesium dietary intake control case

Substituting (C.16) into (C.13) gives

$$\frac{dC_s^*}{dt} = \frac{1}{1+\xi_1+\xi_B\xi_2}\left(\xi\,\Gamma^*(\rho - C_s^*) + \mu(C_N^* - C_s^*) + (1-\xi)(C_T^* - C_s^*) + \xi(C_I^* - C_s^*)\right), \tag{C.44}$$

from which the analysis proceeds in exactly the same way, whereby the composite solutions are identical to (C.41) and (C.42) except that the coefficient of $(1-e^{-t/T^*_{Mg}})$ is $(\xi S/\Gamma\xi_T+\rho-1)$.

In terms of dimensional parameters

$$C_s^* \sim C_N^* \sim C_T^* \;\sim 1 \,+\, \left(\frac{\sigma(1-\xi)}{\phi_D} + \rho - 1\right)\left(1 - e^{-t/T_{Mg}}\right), \tag{C.45}$$

$$C_I^* \;\sim 1 + \frac{\sigma\,\gamma}{k_1\,\phi_D}\frac{V_T}{V_I}\left(1 - e^{-t/T_1}\right) \,+\, \left(\frac{\sigma(1-\xi)}{\phi_D} + \rho - 1\right)\left(1 - e^{-t/T_{Mg}}\right), \tag{C.46}$$

as $\xi \to 0^+$, being the modified version of (6) and (7) and

$$T_{hyp} \;=\; T_{Mg}\ln\left(\frac{\sigma(1-\xi) - (1-\rho)\phi_D}{\sigma(1-\xi) - (C^*_{hyp} - \rho)\phi_D}\right), \tag{C.47}$$

for $\sigma \geq \sigma_{hyp} = (C^*_{hyp} - \rho)\phi_D/(1-\xi) \approx (C_{hyp} - \rho)\phi_D$.

C.3.2 More rigorous formulation

Inspection of the parameters in Table S4 shows that there are several "small" parameters, where in particular Γ, μ, ξ_T and ξ_N can reasonably be considered to be $O(\xi^{1/2}) \approx O(0.01)$ in size, more formally they lie in the range $\xi^{1/4} = O(0.1)$ and $\xi^{3/4} = O(0.001)$. The dimensionless release rate S is borderline and is assumed $S = O(1)$ here (certainly relevant for Mg implants with higher corrosion rates or if larger/multiple devices are used). Writing

$$(\Gamma, \mu, \xi_T, \xi_N) \;=\; \xi^{1/2}\,(\Gamma^*, \mu^*, \xi_T^*, \xi_N^*),$$

where $\Gamma^*, \mu^*, \xi_T^*, \xi_N^*$ are all assumed $O(1)$, and proceeding with the singular perturbation analysis as before, it turns out that there are three notable timescales $t = O(1), O(\xi^{-1/2})$ and $O(\xi^{-1})$. In the first two timescales C_I^* increases (analogous to the first timescale discussed in Section C.3.1.1), and in the third all the state variables increase (analogous to that in Section C.3.1.2). Omitting the details and considering the dietary case for completeness (equivalent to results in Section C.3.1.3) the composite solutions are

$$C_s^* \;\sim\; \rho + \frac{S}{\Gamma^*\xi_T^*} + A_s\,e^{-\omega^-\xi\,t} - \left(\rho - 1 + \frac{S}{\Gamma^*\xi_T^*} + A_s\right)e^{-\omega^+\xi\,t}, \tag{C.48}$$

$$C_N^* \sim \rho + \frac{S}{\Gamma^* \xi_T^*} + A_N\, e^{-\omega^- \xi\, t} - \left(\rho - 1 + \frac{S}{\Gamma^* \xi_T^*} + A_N\right) e^{-\omega^+ \xi\, t}, \tag{C.49}$$

$$C_T^* \sim \rho + \frac{S}{\Gamma^* \xi_T^*} + A_s\, e^{-\omega^- \xi\, t} - \left(\rho - 1 + \frac{S}{\Gamma^* \xi_T^*} + A_s\right) e^{-\omega^+ \xi\, t}, \tag{C.50}$$

$$C_I^* \sim \frac{S(1 - e^{-\beta\sqrt{\xi}\, t})}{\xi^{1/2}\, \xi_T^*} + \rho + \frac{S}{\Gamma^* \xi_T^*} + A_s\, e^{-\omega^- \xi\, t} - \left(\rho - 1 + \frac{S}{\Gamma^* \xi_T^*} + A_s\right) e^{-\omega^+ \xi\, t}, \tag{C.51}$$

as $\xi \to 0^+$, where $\beta = \xi_T^*/(1 + \xi_3)$,

$$A_s = \frac{(S + (\rho - 1)\Gamma^* \xi_T^*)(\omega^+(1 + \xi_3) - \Gamma^* \xi_T^*)}{\Gamma^* \xi_T^* (1 + \xi_3)(\omega^- - \omega^+)}, \quad A_N = \frac{\xi_T^*(\Gamma^* + \mu^*) - (1 + \xi_3)\omega^-}{\mu^* \xi_T^*} A_s$$

and $0 < \omega^- < \omega^+$ are the real roots of a quadratic equation whose solutions are

$$\omega^\pm = \frac{c_1}{2} \pm \frac{(c_1^2 - 4c_0)^{1/2}}{2},$$

with $c_0 = \Gamma^* \mu^* \xi_T^* \xi_N^* / \phi(1 + \xi_3)$ and $c_1 = (\mu^* \xi_N^* (1 + \xi_3) + \phi \xi_T^* (\Gamma^* + \mu^*))/\phi(1 + \xi_3)$; the approximation for the standard model can be derived by simply plugging in $\rho = 1$. We note that ω^- and ω^+ are independent of S, so in common with the simple approximation of Section C.3.1, this analysis predicts that the timescale for systemic Mg elevation is dependent only on the physiologically based parameters and not on the corrosive properties of the implant; furthermore, the equivalent time point to T_{Mg} is $1/\xi\omega^-$ in dimensionless terms, converting to the dimensional form

$$T_{Mg}^{rigour} = \frac{V_s}{k_1\, \xi\, \omega^-}, \tag{C.52}$$

giving $T_{Mg}^{rigour} \approx 121$ days using Table 1. We further note that the dimensionless form of $T_1 = 1/\beta\sqrt{\xi} = (1 + \xi_3)/\xi_T$ is the same as that of the simpler formulation. An explicit formula for T_{hyp}, equivalent to that in Table 2 or (C.47) is not possible in this case, but it can be routinely calculated by solving $C_s^*(T_{hyp}) = C_{hyp}^*$ from (C.48) (note the same constraint, $S > \Gamma^* \xi_T^* (C_{hyp}^* - \rho)$) using Newton-Raphson method, for example.

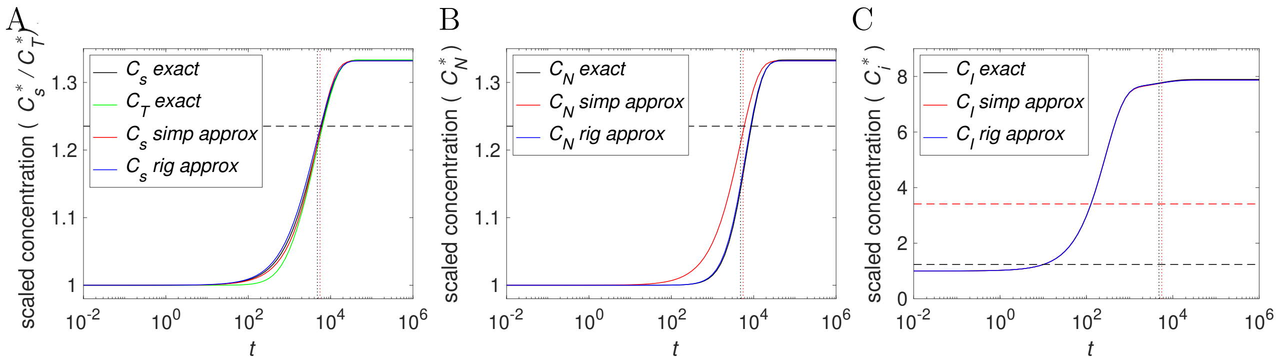

Figure S4: Plots comparing the dimensionless time dependent solutions between the full model (black, green), equations (C.8)-(C.12), "simple approximations" using equations (C.40)-(C.42) (red) and "more rigorous approximations" using equations (C.48)-(C.51) (blue), for the 40 implant case depicted in Fig. 3. The dashed lines indicate hypermagnesemia and problematic levels as in previous models. The vertical dotted lines indicate correspond to the dimensionless time point $t = T_{Mg}$ (black) and T_{Mg}^{rigour} (red). All other parameters are given in Table S4 using the scalings indicated in Sections C.3.1 and C.3.2 noting here that $\xi = 0.004$.

In Fig. 7, there is a slight dip in serum concentration before evolving to the steady-state level ($C_s^* \sim \rho + S/\Gamma^* \xi_T^*$). It turns out that this "dip" emerges in the $t = O(1)$ timescale in the correction term for C_s^*, where it can be shown that

$$C_{s_{min}}^* \sim 1 + \xi^{1/2} (\rho - 1)\Gamma^*; \tag{C.53}$$

as $\xi \to 0^+$, thus predicting only a minor reduction, $O(\xi^{1/2}) \ll 1$, in serum Mg levels in the first two timescales.

Fig. S4 shows comparisons of the exact and approximate solutions for the 40 implant case (used for illustrative purposes). The approximations agree well for C_s^* and C_I^*, though both resulting with $C_T^* = C_s^*$ at leading order is a little inaccurate during the early stage of Mg concentration increase in tissues (green curve in Fig.S4A); the latter suggesting that there will be a visible contribution of the correction term in the expansion for C_T^*. Fig.S4B demonstrates the improvement in approximation using the more rigorous formulation, which captures the lag-time in response of the bone well, whilst the simple formulation predicts a more rapid increase in bone concentration.

References

(1) Jahnen-Dechent, W.; Ketteler, M. Magnesium basics. *Clin. Kidney J.* **2012**, *5*, i3–i14, DOI: 10.1093/ndtplus/sfr163.

(2) Swaminathan, R. Magnesium metabolism and its disorders. *Clin. Biochem. Rev.* **2003**, *24*, 47, PMID: 18568054.

(3) Blaine, J.; Chonchol, M.; Levi, M. Renal control of calcium, phosphate, and magnesium homeostasis. *Renal Physiol.* **2015**, *10*, 1257–1272, DOI: 10.2215/CJN.09750913.

(4) Lee, J.; Han, H.; Han, K.; Park, J.; Jeon, H.; Ok, M.; Seok, H.; Ahn, J.; Lee, K.; Lee, D.; Yang, S.; Cho, S.; Cha, P.; Kwon, H.; Nam, T.; Han, J.; Rho, H.; Lee, K.; Kim, Y.; Mantovani, D. Long-term clinical study and multiscale analysis of in vivo biodegradation mechanism of Mg alloy. *PNAS* **2016**, *113*, 716–721, DOI: 10.1073/pnas.1518238113.

(5) Gupta, R.; Benovic, J.; Rose, Z. The determination of the free magnesium level in the human red blood cell by 31P NMR. *J. Biol. Chem.* **1978**, *253*, 6172–6176, DOI: 10.1016/S0021-9258(17)34595-7.

(6) Millart, H.; Durlach, V.; Durlach, J. Red blood cell magnesium concentrations: analytical problems and significance. *Magnes. Res.* **1995**, *8*, 65–76, PMID: 7669509.

(7) Elin, R. Magnesium metabolism in health and disease. *Dis. Mon.* **1988**, *34*, 166–218, DOI: 10.1016/0011-5029(88)90013-2.

(8) Topf, J.; Murray, P. Hypomagnesemia and hypermagnesemia. *Rev. Endocr. Metab. Disord.* **2003**, *4*, 195–206, DOI: 10.1023/a:1022950321817.

(9) Günther, T. Total and free Mg^{2+} contents in erythrocytes: a simple but still undisclosed cell model. *Magnes. Res.* **2007**, *20*, 161–167, PMID: 17972458.

(10) De Baaij, J.; Hoenderop, J.; Bindels, R. Regulation of magnesium balance: lessons learned from human genetic disease. *Clin. Kidney J.* **2012**, *5*, i15–i24, DOI: 10.1093/ndtplus/sfr164.

(11) Sojka, J.; Wastney, M.; Abrams, S.; Lewis, S. F.; Martin, B.; Weaver, C.; Peacock, M. Magnesium kinetics in adolescent girls determined using stable isotopes: effects of high and low calcium intake. *Am. J. Physiol.* **1997**, *273*, R710–R715, DOI: 10.1152/ajpregu.1997.273.2.R710.

(12) Sabatier, M.; Pont, F.; Arnaud, M.; Turnlund, J. A compartmental model of magnesium metabolism in healthy men based on two stable isotope tracers. *Am. J. Physiol.* **2003**, *285*, R656–R663, DOI: 10.1152/ajpregu.00749.2002.

(13) Avioli, L.; Berman, M. Mg^{28} kinetics in man. *J Appl Physiol.* **1966**, *21*, 1688–94, DOI: 10.1152/jappl.1966.21.6.1688.

(14) Brown, R.; Delp, M.; Lindstedt, S.; Rhomberg, L.; Beliles, R. Physiological parameter values for physiologically based pharmacokinetic models. *Toxicol. Ind. Health* **1997**, *13*, 407–484, DOI: 10.1177/074823379701300401.

(15) Chen, W.-Y.; Cheng, Y.-H.; Hsieh, N.-H.; Wu, B.-C.; Chou, W.-C.; Ho, C.-C.; Chen, J.-K.; Liao, C.-M.; Lin, P. Physiologically based pharmacokinetic modeling of zinc oxide nanoparticles and zinc nitrate in mice. *Int. J. Nanomedicine* **2015**, *10*, 6277, DOI: 10.2147/IJN.S86785.

(16) Schroeter, J.; Nong, A.; Yoon, M.; Taylor, M.; Dorman, D.; Andersen, M.; Clewell III, H. Analysis of manganese tracer kinetics and target tissue dosimetry in monkeys and humans with multi-route physiologically based pharmacokinetic models. *Toxicol. Sci.* **2010**, *120*, 481–498, DOI: 10.1093/toxsci/kfq389.

(17) Wang, J.; Xu, J.; Liu, W.; Li, Y.; Qin, L. Biodegradable magnesium (Mg) implantation

does not impose related metabolic disorders in rats with chronic renal failure. *Scientific Rep.* **2016**, *6*, 26341, DOI: 10.1038/srep26341.

(18) Sato, A.; Shimizu, Y.; Imai, Y.; Mukai, T.; Yamamoto, A.; Miura, C.; Muraki, K.; Sano, Y.; Ikeo, N.; Tachi, M. Initial organ distribution and biological safety of Mg^{2+} released from a Mg alloy implant. *Biomed. Mater.* **2018**, *13*, 035006, DOI: 10.1088/1748-605X/aaa9d5.

(19) Zheng, L.; Zhang, R.; Chen, X.; Luo, Y.; Du, W.; Zhu, Y.; Ruan, Y.; Xu, J.; Wang, J.; Qin, L. Chronic kidney disease: a contraindication for using biodegradable magnesium or its alloys as potential orthopedic implants? *Biomed Mater.* **2024**, *19*, 045023, DOI: 10.1088/1748-605X/ad5241.

(20) Biber, R.; Pauser, J.; Gesslein, M.; Bail, H. Magnesium-Based Absorbable Metal Screws for Intra-Articular Fracture Fixation. *Case Rep. in Orthopedics* **2016**, Article ID 9673174, DOI: 10.1155/2016/9673174.

(21) Zhang, F.; Xu, H.; Wang, H.; Geng, F.; Ma, X.; Shao, M.; Xu, S.; Lu, F.; Jiang, J. Quantitative analysis of near-implant magnesium accumulation for a Si- containing coated AZ31 cage from a goat cervical spine fusion model. *BMC Musculoskelet. Disord.* **2018**, *19*, 105, DOI: 10.1186/s12891-018-2027-5.

(22) Varga, R. *Geršgorin and His Circles*; Berlin: Springer-Verlag, 2004; DOI: 10.1007/978-3-642-17798-9.

(23) Johnson, R. *Singular perturbation theory: mathematical and analytical techniques with applications to engineering*; Springer, Berlin, 2006; DOI: 10.1007/b100957.

(24) Miller, J., Ed. *Singular perturbation problems in chemical physics: analytical and computational methods*; Advances in Chemical Physics; John Wiley and Sons, New York, 1997; Vol. 97; DOI: 10.1002/9780470141564.
